# *N*^6^-Methyladenosine Landscape of Glioma Stem-Like Cells: METTL3 Is Essential for the Expression of Actively Transcribed Genes and Sustenance of the Oncogenic Signaling

**DOI:** 10.3390/genes10020141

**Published:** 2019-02-13

**Authors:** Abhirami Visvanathan, Vikas Patil, Shibla Abdulla, Jörg D. Hoheisel, Kumaravel Somasundaram

**Affiliations:** 1 Department of Microbiology and Cell Biology, Indian Institute of Science, Bangalore 560012, India; abhrm_12@yahoo.com (A.V.); patil.vikas1010@gmail.com (V.P.); shiblaabdulla7@gmail.com (S.A.); 2 Functional Genome Analysis, Deutsches Krebsforschungszentrum (DKFZ), Im Neuenheimer Feld 580, 69120 Heidelberg, Germany; j.hoheisel@dkfz-heidelberg.de

**Keywords:** METTL3, m^6^A, GSCs, glioblastoma, histone activation, splicing, RNA editing, m^6^A reader

## Abstract

Despite recent advances in *N*^6^-methyladenosine (m^6^A) biology, the regulation of crucial RNA processing steps by the RNA methyltransferase-like 3 (METTL3) in glioma stem-like cells (GSCs) remains obscure. An integrated analysis of m^6^A-RIP (RNA immunoprecipitation) and total RNA-Seq of METTL3-silenced GSCs identified that m^6^A modification in GSCs is principally carried out by METTL3. The m^6^A-modified transcripts showed higher abundance compared to non-modified transcripts. Further, we showed that the METTL3 is essential for the expression of GSC-specific actively transcribed genes. Silencing METTL3 resulted in the elevation of several aberrant alternative splicing events. We also found that putative m^6^A reader proteins play a key role in the RNA stabilization function of METTL3. METTL3 altered A-to-I and C-to-U RNA editing events by differentially regulating RNA editing enzymes ADAR and APOBEC3A. Similar to protein-coding genes, lincRNAs (long intergenic non-coding RNAs) with m^6^A marks showed METTL3-dependent high expression. m^6^A modification of 3′UTRs appeared to result in a conformation-dependent hindrance to miRNA binding to their targets. The integrated analysis of the m^6^A regulome in METTL3-silenced GSCs showed global disruption in tumorigenic pathways that are indispensable for GSC maintenance and glioma progression. We conclude that METTL3 plays a vital role in many steps of RNA processing and orchestrates successful execution of oncogenic pathways in GSCs.

## 1. Introduction

Glioma stem-like cells (GSCs) represent a primitive form in the hierarchy of tumor cells, which can initiate and populate the tumor. GSCs are highly tumorigenic and contribute to the poor therapy response of glioblastoma (GBM) patients [1]. Dissecting the cellular mechanisms that sustain GSC growth is essential for identifying therapeutic targets. A GSC is a flexible cellular system that undergoes differentiation (differentiated glioma cell, DGC) in the presence of serum and can revert to stem-like cells through reprogramming/dedifferentiation [2]. In addition, GSCs have the potential to undergo inter-conversion of cell state with extra-cellular cues and stress conditions, such as drug treatment [3].

The m^6^A epitranscriptome regulates the expression of a plethora of genes by altering various steps of RNA metabolism, such as mRNA stability, RNA export, alternative polyadenylation, splicing and translatability. The *N*^6^-methyladenosine (m^6^A) modification is facilitated by two methylases (i.e., methyltransferase-like 3 (METTL3) and methyltransferase-like 14 (METTL14)), while demethylation is mediated by alkB homolog 5 (ALKBH5) and fat mass and obesity-associated protein (FTO) [4,5,6]. The dynamic and reversible nature of the m^6^A modification makes it instrumental in rapid cellular response and in creating expression gradients. The epitranscriptome marks act as loading sites for various RNA binding proteins, which decode the modifications into cellular functions. We recently reported that m^6^A modification is elevated in GSCs and dependent on high levels of METTL3, as seen in GSCs compared to DGCs [7]. In addition, reports on the METTL3/METTL14 complex provide support that only METTL3 has a catalytic domain for S-adenosyl methionine binding and shows a non-redundant methylation function [8]. The inhibition of METTL3 in different cell types leads to induced apoptosis, growth arrest, altered stem cell phenotype and aberrant mRNA processing. Our previous study demonstrated that METTL3 mediates global yet specific m^6^A modifications of transcripts that are crucial for stem cell maintenance and the radioresistance of GSCs [7].

Based on these facts, we investigated the METTL3-mediated m^6^A regulome in GSCs by m^6^A RNA immunoprecipitation (RIP) sequencing combined with transcriptome analysis after METTL3 silencing. Herein, we present the impact of METTL3 silencing on global m^6^A modification, transcriptome, various RNA processing steps and important functional pathways. Collectively, our study uncovered the crucial collaborative functions of METTL3-dependent m^6^A modification in RNA metabolism including RNA stability specific for GSCs.

## 2. Materials and Methods

### 2.1. Neurosphere Cell Culturing

The primary tumor GSC, MGG8, was a kind gift of Dr. Wakimoto (Massachusetts General Hospital, Boston, MA, USA) and was cultured as spheres in serum-free neural stem cell medium (Neurobasal medium; Invitrogen, Carlsbad, CA, USA) supplemented with 3 mM l-glutamine (Invitrogen), 1× B27 supplement (Invitrogen), 0.5× N-2 supplement (Invitrogen), 2 µg/mL heparin (Sigma, St. Louis, MO, USA), 20 ng/mL recombinant human EGF (Promega, Madison, WI, USA), 20 ng/mL recombinant human FGF2 (Promega) and penicillin/streptomycin.

### 2.2. RNA Isolation, Reverse Transcription and qPCR Analysis

Total RNA was isolated using TRI reagent (Sigma). Two micrograms of RNA were reverse transcribed with the high-capacity cDNA reverse transcription kit (Life Technologies, Carlsbad, CA, USA) according to the manufacturer’s protocol. Quantitative RT-PCR was performed using the ABI PRISM 7900 HT Sequence Detection System (Life Technologies). Expression of the genes of interest was analyzed using ATP5G rRNA as internal control genes and the ΔΔCt method. Real-time primer information is provided in the Supplementary Materials.

### 2.3. RNA Preparation for RIP-Sequencing

PolyA-RNA was isolated using the Genelute Direct RNA kit (#DMN10, Sigma). Briefly, GSC pellets were lysed and filtered through a column. RNase was eliminated during a 10-min proteinase K digestion. Sodium chloride was added, and polyadenylated RNA captured on oligo-dT-covered polystyrene beads during a 10-min incubation. After three washes in a spin column, purified mRNA was eluted in 100 μL of 10 mM Tris-HCl, pH 7.4. The RNA was subjected to 100bases fragmentation using NEB fragmentation buffer (#E6150S, NEB, Ipswich, MA, USA) for 5 min. The RNA was precipitated using sodium acetate and ethanol. RNA quality was analyzed on a Bioanalyser2100 (Agilent Tech, Santa Clara, CA, USA).

### 2.4. m^6^A RNA Immunoprecipitation Enrichment

Epimark m^6^A enrichment kit (E1610S, NEB) was used according to the manufacturer’s instructions to perform m^6^A RIP. An amount of 1.25 µg purified RNA was added to the magnetic beads, to which an m^6^A-specific antibody was coupled. Followed by low and high salt washes, the bead-bound RNA was directly isolated by the Trizol method. In this step, the beads were divided into two aliquots; elution was performed independently for the purpose of producing technical duplicates. The quality of RNA was analyzed on a Bioanalyser 2100 (Agilent Tech) and the RNA concentration was quantified with a Qubit RNA HS kit (#Q32852, Invitrogen).

### 2.5. m^6^A RNA Immunoprecipitation Sequencing

A library was prepared from each RNA sample. Five nanogram of each m^6^ARNA immunoprecipitation (RIP) sample were used. As a control, a library was prepared also from 10ng of the respective polyA-RNA sample used for m^6^A RIP enrichment. The NEBNext RNA Ultra II library prep kit (#E7770S, NEB) was applied and samples were prepared according to the manufacturer’s instruction with slight modifications. The fragmentation step was omitted and the fragments were purified using QIAQuick column (#28104, Qiagen, Hilden, Germany) after second-strand synthesis. Index primers of NEBNext Multiplex oligonucleotides for Illumina (Index Primers Set 1) were used (E7335S, NEB). The final PCR enrichment of adaptor-ligated libraries was performed in 12 cycles. Library quality was confirmed on a Tapestation2200 (Agilent Tech). The DNA concentration was quantified with the Qubit dsDNA HS kit (Q32851). Volumes corresponding to 2 nmol DNA were pooled and sequenced on a HiSeq2000 (Illumina) with 50bases single-end read.

### 2.6. Transfection of Cells with Plasmids and siRNAs

For knockdown, either METTL3 small interfering RNA (On-target plus SMART Pool, Dharmacon, Lafayette, CO, USA) or a non-targeting siRNA control (siNT) were transfected using Dharmafect I (Dharmacon) according to the manufacturer’s instructions. The shRNA construct for METTL3 (TRCN0000289814) was taken from the MISSION shRNA TRC whole genome library.

For overexpression, a *METTL3* construct in pCMV-Entry vector (#RC200869, Origene, Rockville, MD, USA) was used; transfection of the empty vector acted as a control. Plasmids were transfected using Lipofectamine 2000 (Thermo Fisher Scientific, Waltham, MA, USA) according to the manufacturer’s instructions. After 48 h, cells were harvested and transfection with the correct construct was confirmed by qRT-PCR. Simultaneously, cells were split into single cell suspension and counted; equal cell numbers were plated.

### 2.7. RNA Stability Assay

Actinomycin D (#A9415, Sigma) at 5 μg/mL was added to cells 24 h after transfection of siNT and siMETTL3. After 3 and 6 h incubations, cells were collected and RNA was isolated for RT-qPCR. GAPDH was used for normalization.

### 2.8. Western Blot

For Western blot analysis, RIPA buffer was used to isolate protein lysates from the cell lines. The protein concentration was quantified by Bradford’s reagent (Biorad, Hercules, CA, USA). The following antibodies have been used in these studies: Anti-METTL3 (Cat.No.H00056339-B01P, Abnova, Taipei, Taiwan), Anti-Notch1 (Cat.No.C-20, Santa Cruz, TX, USA), Anti-Hes-1(Cat.No.H-20, Santa Cruz) and Anti-Actin (Cat.No.A5316, Sigma).

### 2.9. RNA Immunoprecipitation of METTL3

Cells stably expressing control ENTRY vector and METTL3/DDK/MYC vector were harvested in polysome lysis buffer and frozen at −80 °C to maximize the lysis efficiency. The lysates were cleared by centrifugation at 14,000 rpm and the amount of protein in the supernatant was quantified by Bradford’s Reagent (Biorad). Buffer equilibrated protein G agarose beads (Sigma) were incubated with equal amount of METTL3 antibody (Abnova) and IgG control antibody (CST) for 6 h. Following washing, the lysates were incubated over night with antibody bound beads at 4 °C. The RNA was eluted with TRI reagent (Sigma) and converted to cDNA by using a cDNA Synthesis kit (ABI Prism, Waltham, MA, USA). Equal volumes of cDNA were used for RT-qPCR for quantification of the fold enrichment.

### 2.10. Luciferase Reporter Assay

In total, 10^5^ cells were plated on a 12-well microtiter plate and co-transfected using 2 μg of HES1-Luc and 0.25 μg β-galactosidase coding plasmid. Cells were harvested after 24 h. Cell lysates were prepared using reporter lysis buffer (#E3971, Promega) and a luciferase assay was performed with equal amounts of protein using a luciferase assay reagent (#E1483, Promega) in a luminometer (Berthold, Bad Wildbad, Germany). For transfection normalization, a β-galactosidase assay was performed.

### 2.11. RNA Isolation, cDNA Synthesis and qRT-PCR

TRI reagent (Sigma) was used to isolate total RNA from pellets of shNTorshMETTL3 transduced cells according to the manufacturer’s instructions. The RNA quantity and quality were checked in a NanoDrop instrument (ND-1000 Spectrophotometer, Thermofisher, Waltham, MA, USA) and 2% denaturing gels containing MOPS-formaldehyde, respectively. Two micrograms of total RNA were used for cDNA synthesis using a conversion kit (#4352405, Applied Biosystem, Waltham, MA, USA) in a Biorad S1000 Thermal Cycler. qRT-PCR was performed in an ABI 7900HT real-time machine using the Dynamo master mix (Applied Biosystem). Transcript levels were analyzed using ATP5G as internal control and ΔΔCT method. The real-time primers used are listed in Table 1.

### 2.12. Lentivirus Preparation

HEK293T cells were grown in a 60 mm dish coated with poly-l-lysine solution (P4832, Sigma) and transfected with shRNA construct along with helper plasmids—psPAX2 and pMD2.G (Addgene, Watertown, MA, USA)—using Opti-MEM reduced serum medium (#22600050, Gibco, Waltham, MA, USA) and Lipofectamine 2000 (#11668500, Invitrogen). The growth medium was replaced with DMEM (containing 10% FBS) 6 h after transfection. The supernatant was collected after 60 h. The supernatant was centrifuged at 5000 rpm for 10 min and aliquots were stored at 0°C.

### 2.13. RNA Editing

RNA-Seq data were used to identify RNA editing events following a rigorous and robust analysis pipeline [9]. The RNA-seq reads were aligned to the human reference genome (hg19) and transcriptome (Ensembl64) using the Burrows–Wheeler Aligner version 0.5.7 [10]. Those reads mapped to multiple locations (genomic and transcriptomic) were collapsed into single genomic coordinates using the Remap Transcriptome tool of GATK [11]. For co-ordinate sorting and duplicate removal, Picard was used (http://broadinstitute.github.io/picard/). Read re-alignment and base recalibration were done using GATK [11]. Next, variants were called using GATK’s Unified Genotyper with options stand_call_conf of 0 and stand_emit_conf of 0. The total variants obtained were then filtered to remove potential polymorphisms by comparing with three databases: dbSNP (http://www.ncbi.nlm.nih.gov/SNP/), the 1000 genomes database (http://www.internationalgenome.org/), and ESP6500 (http://evs.gs.washington.edu/EVS). The first six bases of each read were discarded to remove artificial mismatches caused by random-hexamer priming. The editing events present in non-Alu regions were then subjected to further selection steps filtering out spurious changes: (1) each editing event was to be represented by at least 3 reads containing the altered nucleotide and the minimum frequency of the altered nucleotide had to be 0.1; (2) any site present in simple repeats was removed; (3) any candidate change present within 4 bp of known splice junctions was removed; (4) sites present in homopolymer runs of ≥5 bp were removed; and (5) sites located in regions having a high similarity to sequences present in other parts of the genome (found out by using BLAST) were removed. Editing events for which 10 or more reads were found and the editing ratio was ≥0.2 were considered as high confidence editing events. Further, hg19 genome coordinates were converted to hg38 using the hgLiftOver utility of the UCSC genome browser (https://genome.ucsc.edu/cgi-bin/hgLiftOver).

### 2.14. Differential Alternative Splicing

We used rMATS (replicate Multivariate Analysis of Transcript Splicing) [12] to identify differential alternative splicing events in cells transduced with non-targeting shRNA (shNT) or METTL3 shRNA (shMETTL3). rMATS shows skipped exon (SE), alternative 5′ splice site (A5SS), alternative 3′ splice site (A3SS), mutually exclusive exon (MXE), and retained intron (RI) events. We aligned RNA-Seq reads to the human reference genome hg38 using STAR aligner [13]. We used BAM file and gencode GTF file (gencode version 25) (https://www.gencodegenes.org/) as input to rMATs with an inclusion-level difference ≥5 and events with a FDR < 0.05 cut-off in order to determine differential alternative splicing. Splicing events having inclusion counts and skipping counts ≥ 10 in both conditions (shNT and shMETTL3) were considered for further analysis.

### 2.15. Alignment of RNA-Sequences

The quality assessment of the raw reads was carried out using the FastQC tool. The reads were aligned to the human reference genome, hg38, i.e., the Human Genome Reference Consortium build 38 (GRCh38) using the TopHat2 aligner [14]. The duplicate removal was carried out using Picard 1.73. The RNA-Seq reads were counted over gene exons using HtSeq. Genes were annotated as per the Gencode Version 25 annotation file (http://www.gencodegenes.org/releases/25.html). DEseq2 [15] was used to identify the differentially expressed genes between the shNT_GSC and shMETTL3_GSC samples with an adjusted *p*-value cutoff of 0.05 and an absolute fold change >1.5. Data accompanying this paper is available through SRA accession number SRP163326.

### 2.16. Gene Set Enrichment Analysis (GSEA)

The computational approach of gene set enrichment analysis (GSEA) [16] was undertaken to evaluate the enrichment of specific pathways or gene sets in a given input of regulated transcriptome. Pre-ranked GSEA was performed with default parameters (1000 sample permutations). The genes which were significantly regulated (absolute fold change >1.5)) by METTL3 silencing compared to control condition with significant *p*-value were ranked according to the expression changes and represented the input genes. Hallmark pathways of cancer were chosen as gene set and GSEA was performed using default parameters. For identifying the GSC-specific gene enrichment, we used genes which were regulated by 0.58 > log_2_ ratio with a significant *p*-value (<0.05) in the GSE54791 dataset (GSC-vs. DGC-reconstructing and reprogramming the tumor propagating potential of glioblastoma stem-like cells: RNA-Seq).

The datasets used for histone modification related GSEA were GSE46014 (Histone modification and TF ChIP-Seq for glioblastoma cell lines and neural stem cells) for H3K4me3/H2K27me3 in MGG8 GSC compared to NSC or DGC, and GSE54047 (Reconstructing and reprogramming the tumor propagating potential of glioblastoma stem-like cells: ChIP-Seq) for H3K27ac in MGG8 GSC compared to MGG8 DGC. Three comparisons were performed:

1. Genes were grouped as activated (H3K4me3) or repressed (H3K27me3, H3K27me3 + H3K4me3) in MGG8 GSC compared to NSC.
2. Genes were grouped as activated (H3K4me3) or repressed (H3K27me3, H3K27me3 + H3K4me3) in MGG8 GSC compared to MGG8 DGC.
3. Genes were grouped as activated (H3K27Ac) in MGG8 GSC compared to MGG8DGC.

These genes were analyzed for up/downregulation at transcript level (0.58 > log_2_ ratio) with significant *p*-value (<0.05) in GSC compared to NSC (normal neural stem cells) or DGC (differentiated glioma cells) from GSE45899 (Expression profiling of glioblastoma cancer stem cells-microarray) and GSE54791 (GSC vs. DGC - –Reconstructing and reprogramming the tumor propagating potential of glioblastoma stem-like cells: RNA-Seq), respectively.

After merging the correlating gene lists, the three following gene sets were made:

1. The activated and upregulated/repressed and downregulated genes in GSC compared to NSC based on H3K27me3/H3K4me3marks.
2. The activated and upregulated/repressed and downregulated genes in GSC compared to DGC based on H3K27me3/H3K4me3marks.
3. The activated and upregulated genes in GSC compared to DGC based onH3K27Ac

The input genes for the GSEA analysis were all regulated genes after METTL3 silencing (0.58 log_2_ ratio with significant *p*-value of < 0.05) or direct METTL3 targets (genes regulated by 0.58 log_2_ ratio with significant *p*-value of < 0.05 andMETTL3 dependent m^6^A peaks). The ranked list based on expression regulation was given as input for GSEA.

### 2.17. Pathway Analysis

The Database for Annotation, Visualization and Integrated Discovery (DAVID) Bioinformatics Resources tool version 6.7 [17] was used to identify the enriched Panther and Reactome pathway categories for direct/indirect target genes and splice variants with a *p*-value cutoff of less than 0.05. GO analysis was performed for the Top 50 m^6^A bound RNA binding proteins identified by RBPmap [18] (http://rbpmap.technion.ac.il/).

### 2.18. RNA Immunoprecipitation Sequencing Analysis

#### 2.18.1. Alignment of m^6^A RNA Immunoprecipitation Peaks

The sequencing output in the form of base intensity files was converted to the fastq format and subsequently demultiplexed using bcl2fastq. Next, reads were aligned to the human reference genome hg38 and the human reference transcriptome GencodeV25 by TopHat2 (version 2.1.1). PCR duplicates were removed using Picard (version 1.73). To avoid interference of introns in peak calling, aligned reads were converted into isoform-based coordinates from genome-based coordinates.

#### 2.18.2. Peak Calling for m^6^A Samples

For peak calling, we scanned a window of 100 nucleotides with a 50 nucleotide overlap and calculated the mean coverage of each window for the immunoprecipitated and input control samples (Mean Win IP and Mean Win Control). We also calculated gene median coverage for immunoprecipitation and input control (Median Gene IP and Median Gene Control, respectively). Fold enrichment of immunoprecipitated over input control samples for every window was calculated by the following formula:

WinScore = log_2_ ((MeanWinIP/MedianGeneIP)/(MeanWinControl/MedianGeneControl))

A *p*-value was calculated for each window with detected enrichment above a defined cutoff, using a Fisher’s exact test. *p*-values were then subjected to Benjamini–Hochberg correction. Consecutive significantly enriched windows were then merged together. We used a *p*-adjusted cut-off of less than 0.05. Data accompanying this paper is available through SRA accession number SRP163326.

#### 2.18.3. Peak Visualization

To make read coverage tracks, we calculated the m^6^A-IP enrichment value at each nucleotide position. We first normalized the read coverage at each nucleotide position to 1 million reads for m^6^A-immunoprecipitated and its input. We used Integrative Genomics Viewer (IGV version 2.3.94) for visualizing selected genes.

#### 2.18.4. Distribution of m^6^A Peak Regions in 5′UTR, exon and 3′UTR

We used the GencodeV25 gene annotation file. We took only the longest transcript isoform of each mRNA. We classified genes into 3′UTR, exon and 5′UTR region. Distribution of all peaks was normalized to the length of this region and plotted using R software (version 3.2.3).

### 2.19. Cumulative Frequency Distribution

Cumulative frequency distribution graphs were generated using R software (version 3.2.3).

### 2.20. miRNA Target Prediction

Peaks present in the 3′UTR of protein coding genes were taken and nucleotide sequences for these peaks were fetched. The TargetScan algorithm [19] was used to predict targets of miRNA for these peaks. We used the context+ score; cut-off was ≤−0.4. Further, we selected genes that were 5× log_2_ downregulated in shMETTL3 condition as compared to shNT. We used Cytoscape (version 3.3.0) [20] for visualizing the network.

### 2.21. Motif Finding

De novo motif analysis was done using the MEME (version 4.12.0) tool [21]. m^6^A peaks present in protein coding genes were chosen for de novo motif finding analysis. Fifty nucleotide sequences were taken from upstream and downstream of the peak summit.

## 3. Results

### 3.1. Transcriptome-Wide Mapping of m^6^A Modification Landscape in Glioma Stem-Like Cells

**Figure 1:**
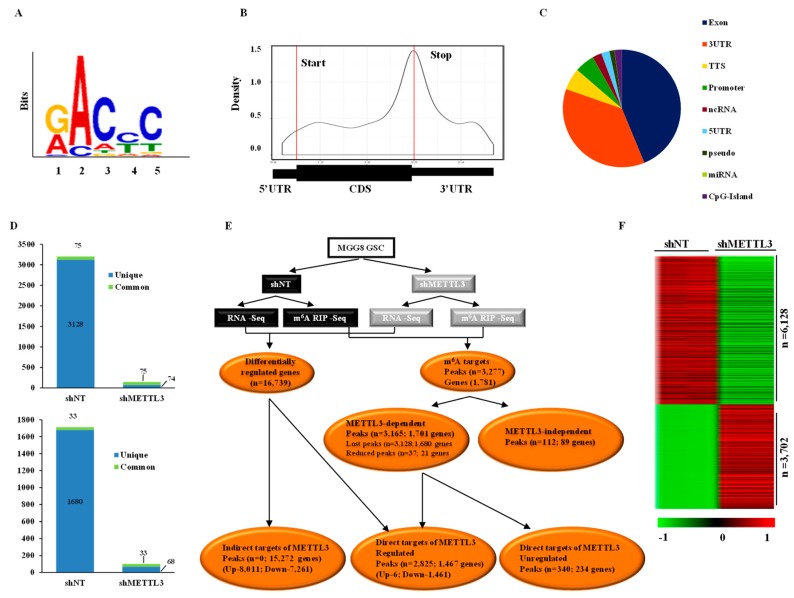
Methyltransferase-like 3 (METTL3)-dependent *N*^6^-methyladenosine (m^6^A) peak distribution and transcriptome regulation in glioma stem-like cells (GSCs). (**A**) A consensus sequence for m^6^A modification was constructed from the Top 1000 m^6^A peaks using MEME (www.meme-suite.org). (**B**) m^6^A peak distribution was plotted along a normalized transcript composed of three rescaled segments of 5′UTR, exon and 3′UTR. Peaks were strongly enriched near the stop codon, which extended into the 3′UTR. (**C**) The distribution of m^6^A peaks in non-targeting shRNA (shNT)-GSCs across different RNA functional regions. (**D**) The number of peaks and genes identified in shNT and METTL3 shRNA (shMETTL3) m^6^A RNA immunoprecipitation (RIP) samples. The number of peaks (left panel) and corresponding genes (right panel) which were lost after METTL3silencing are shown. A global inhibition of m^6^A modification was observed in METTL3-silenced cells. (**E**) An integrated analysis representation of differential m^6^A peaks and transcripts post-METTL3-silencing. Genes which were altered post-METTL3-silencing at the RNA level and had lost or reduced peaks in shMETTL3-GSCs were direct targets regulated at the transcript level. Genes that showed differential regulation with METTL3silencing without m^6^A peaks were considered indirect targets. The number of peaks and corresponding number of genes are shown for each group. Direct targets were divided into two groups based on differential RNA regulation. (**F**) Heat map of differentially regulated protein-coding genes (indirect target) between shNT-GSCs and shMETTL3-GSCs in duplicates. Red indicates high and green low expression with the log_2_ ratio scale of 1 to −1.

To understand the role of METTL3-mediated m^6^A modification in GSCs, we employed an integrated approach of whole-transcriptome and m^6^A RNA-immunoprecipitation-coupled sequencing (m^6^A-RIP Seq) of RNA isolated from MGG8 GSCs transduced with a non-targeting shRNA (shNT) or METTL3 shRNA (shMETTL3). We mapped the m^6^A peaks after normalizing to transcript abundance in shNT-MGG8 and shMETTL3-MGG8 GSCs to identify the METTL3-dependent m^6^A modifications. This analysis identified 3203 m^6^A peaks corresponding to 1713 genes in shNT-MGG8 GSCs and 149 m^6^A peaks from 101 genes in shMETTL3-MGG8 GSCs (Tables S1 and S2). A search for a consensus motif identified “GACPyC” to be enriched in m^6^A sites, reaffirming the previously identified m^6^A motif “RRAC” (Figure 1A). We found that the m^6^A peaks were present throughout the genes but with a distinct enrichment around the stop codons that also extended into the 3′UTR (Figure 1B). The distribution of m^6^A peaks across different gene segments is shown in Figure 1C. Further, we found that a majority of m^6^A-modified genes (over 50%) had one peak per gene (Figure S1A). We also found no significant correlation between the m^6^A peak enrichment score and the number of m^6^A consensus sites contained within a peak, thus emphasizing the fact that the modification may require a specific secondary structure in addition to the mere presence of consensus sequence (Figure S1B). From these results, we conclude that m^6^A modification occurs in specific genes and is particularly enriched around the stop codon, extending into the 3′UTR and could have a possible regulative effect on RNA abundance.

Upon METTL3 silencing, we observed a global decrease in m^6^A levels in GSCs, thus confirming that m^6^A modification is primarily mediated by METTL3 (Figure 1D). In the METTL3-silenced condition, 3128 peaks in 1680 genes were completely lost compared to the shNT condition (unique to METTL3). There were also 75 peaks (33 genes) common to both shNT and shMETTL3 conditions (Figure 1D). Further analysis revealed that the decrease in m^6^A peaks in the METTL3-silenced condition occurred across all gene segments with a maximum reduction observed in the 3′UTR (Figure S1C). This suggests that METTL3 and the associated m^6^A modification may work through the 3′UTR for regulation of RNA metabolism. We mapped the peak density of global m^6^A modification to the exact position, revealing enrichment near 60 base pairs upstream of the stop codon (Figure S1D). These results suggest that m^6^A modification in GSCs is primarily mediated by METTL3 and further reveal that m^6^A modification regulates RNA metabolism by targeting the 3′UTR.

### 3.2. Impact of METTL3-Mediated m^6^A Modification on the Glioma Stem-Like Cells Transcriptome

An integrated analysis of m^6^A RIP-Seq and RNA-Seq revealed several interesting facts (Figure 1E). We found a total of 16,739 RNAs to be regulated in the shMETTL3 condition (Figure S1E). While the protein-coding RNAs constituted the majority of the regulated RNAs (67.05%), there was a significant number of other RNA types, such as lncRNAs and anti-sense RNAs, that were also regulated (Figure S1E). m^6^A RIP-Seq analysis revealed the presence of 3203 m^6^A peaks corresponding to 1713 transcripts in shNT-MGG8 GSCs. We found that the majority of m^6^A peaks seen in shNT-MGG8 GSCs were METTL3-dependent as they either showed a complete loss (peaks *n* = 3128; genes *n* = 1680) or reduced intensity (peaks *n* = 37; genes *n* = 21) in METTL3-depleted GSCs, which suggests that the majority of m^6^A-modified transcripts GSCs are METTL3 direct targets (Figure 1E). We also found a small percentage of METTL3-independent m^6^A peaks (*n* = 112; 89 genes), which were found in shMETTL3-MGG8 GSCs alone or reduced in shNTGSCs compared to shMETTL3 GSCs. Among the transcripts that contained METTL3-dependent m^6^A peaks, the majority of the genes were downregulated at the transcript level in METTL3-silenced GSCs (*n* = 1467; 86.24%). Interestingly, further analysis revealed that almost all regulated genes were actually downregulated (*n* = 1461; 99.6%). There was a small cohort of transcripts that contained METTL3-dependent m^6^A peaks (*n* = 234; 13.8%) that were not regulated at the RNA level upon METTL3 silencing, emphasizing that other RNA metabolic steps of these genes may be fine-tuned by METTL3. Among the direct targets of METTL3, the transcript downregulation showed negative correlation with the number of peaks per gene (Figure S1F).

Further investigation of the METTL3-regulated transcriptome revealed that a majority of regulated transcripts (*n* = 15,272; 91.24%) were identified as indirect targets of METTL3 as these transcripts did not carry m^6^A modification (Figure 1E). Unlike the direct targets of METTL3, the indirect targets showed both upregulation (*n* = 8011; 52.46%) and downregulation (*n* = 7261; 47.5%) at similar levels. However, when we specifically analyzed the protein-coding genes (*n* = 9830) among the indirect targets of METTL3, we found that a larger subset (*n* = 6128; 62.34%) was downregulated compared to the number of upregulated genes (*n* = 3702; 37.7%) in the METTL3-silenced condition (Figure 1F and Table S3). These results together demonstrate that METTL3-mediated m^6^A modification is essential for maintaining the level of m^6^A-modified transcripts. Further, METTL3 also regulates a large number of indirect targets. We thus conclude that METTL3 mediates its function by positively regulating its direct targets, which in turn regulate a larger transcriptome.

### 3.3. METTL3 Is Essential for the Expression of Epigenetically Activated Genes in Glioma Stem-Like Cells

**Figure 2:**
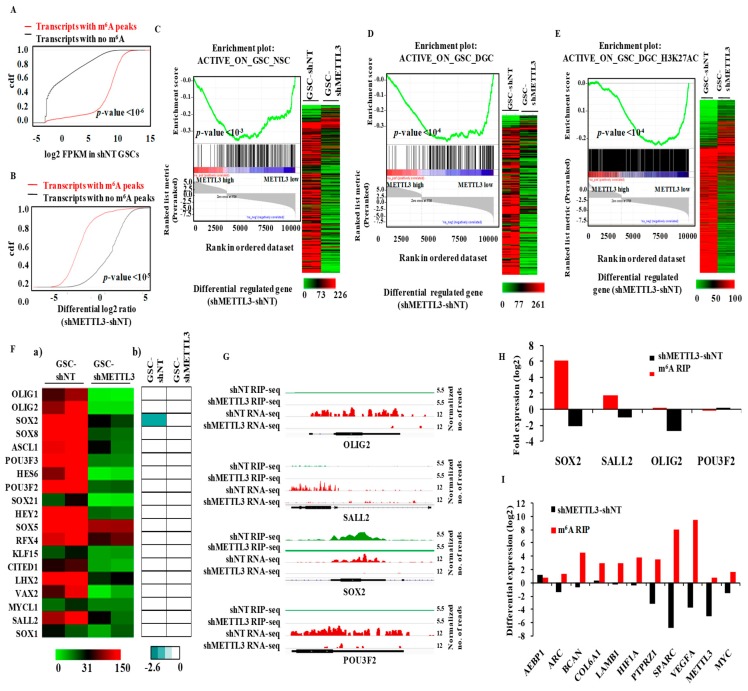
Functional coordination of direct METTL3 targets with epigenetic/transcriptional activation. (**A**) The abundance of m^6^A-modified transcripts was compared with non-modified transcripts. FPKM (fragments per kilobase of transcript per million mapped reads) of each group was shown as cumulative fraction. m^6^A modified transcripts were high in expression. (**B**) The cumulative distribution of fold change (shMETTL3-shNT) among methylated transcripts and unmethylated genes was compared. Methylated targets were significantly inhibited by METTL3silencing compared to unmethylated genes (*p*-value = 0.00001). (**C**) (Left panel) Gene set enrichment analysis (GSEA) enrichment plot showed a depletion of the GSC-specific transcriptionally activated gene set (ACTIVE_ON_GSC_NSC) compared to Neuronal stem cells (NSC) in METTL3-regulated targets (shMETTL3-shNT). The active gene set contained genes with H3K4me3 (active mark) in their promoters. (Right panel) The normalized read counts of genes (shNT-GSC and shMETTL3-GSC) that were enriched for the ACTIVE_ON_GSC_NSC gene set are depicted as heatmap. Red and green denotes high and low expression, respectively. The majority of genes that are activated by active histone mark in GSCs were downregulated in METTL3-silenced condition. (**D**) (Left panel) GSEA enrichment plots showing a depletion of the GSC-specific transcriptionally activated gene set (ACTIVE_ON_GSC_DGC) compared to Differentiated glioma cells (DGC) in METTL3-regulated targets (shMETTL3-shNT). The active gene set contained genes with H3K4me3 (active mark) in their promoters. (Right panel) The normalized read counts of genes (shNT-GSC and shMETTL3-GSC) that were enriched for the ACTIVE_ON_GSC_DGC gene set are depicted as heat map. Red and green denotes high and low expression, respectively. The majority of genes that are activated by active histone mark in GSCs were downregulated in METTL3-silenced condition. (**E**) (Left panel) GSEA enrichment plots showing a depletion of the GSC-specific transcriptionally activated gene set (ACTIVE_ON_ GSC_DGC_H3K27AC) compared to DGC in METTL3-regulated targets (shMETTL3-shNT). The active gene set contained genes with H3K27Ac (active mark) in MGG8 GSCs but not in MGG8 DGCs. (Right panel) The normalized read counts of genes (shNT-GSC and shMETTL3-GSC) that were enriched for ACTIVE_ON_ GSC_DGC_H3K27AC were depicted as heat map. Red and green denotes high and low expression, respectively. The majority of genes that are activated by active histone mark in GSCs were downregulated in METTL3-silenced condition. (**F**) Heat maps are shown depicting:(a) normalized read counts in shNT RNA-Seq and shMETTL3 RNA-Seq; and(b) the m^6^A enrichment score obtained in shNT RIP-Seq and shMETTL3 RIP-Seq for 19 TFs identified [2] as reprogramming factors in glioma cells. High and low expression values (log_2_) are denoted by red and green, respectively. High m^6^A peak enrichment is represented by aqua green and the absence of a peak is depicted by white. (**G**) The genomic tracks of reprogramming transcription factors (TFs) SOX2, SALL2, POU3F2, and OLIG2 are depicted in shNT and shMETTL3-GSCs. Normalized sequence coverage of m^6^A RIP peaks and RNA-Seq reads is indicated above the gene architecture in UCSC format. Thin black boxes represent the 5′ and 3′UTRs; thick black boxes represent the coding sequences; and thin lines represent introns. Among the four, only SOX2 showed m^6^A RIP peaks in control GSCs, which was depleted in shMETTL3 GSCs. (**H**) m^6^A enrichment and transcript level was measured for four reprogramming factors by m^6^A-RIP-PCR and RT-qPCR. The enrichment was calculated compared to IgG Ct values. (**I**) m^6^A enrichment in shNT-GSCs and transcript level changes with METTL3-silencing in MGG8 GSCs was measured by real-time PCR. The enrichment was calculated compared to IgG Ct values. The ddCt value of differential expression was calculated by normalizing with ATP5G.

Since our results show that most m^6^A-modified transcripts require m^6^A modification for their stability, we next investigated the connection between transcript abundance and m^6^A modification. An analysis was performed in consideration of the global RNA population, and the m^6^A-modified RNAs showed high abundance compared to non-modified RNAs (Figure 2A). We further found that the direct targets of METTL3 (i.e., those having m^6^A peaks) were downregulated in the METTL3-silenced condition compared to transcripts with no m^6^A modification (Figure 2B), emphasizing the importance of METTL3-mediated m^6^A modification to the observed high abundance of m^6^A-modified transcripts. Based on these results (i.e., that m^6^A modification has an RNA stabilization function and METTL3-dependent m^6^A modification is essential for the functions of GSCs [7]), we investigated the impact of this process on the epigenetic program that maintains the GSC phenotype. The maintenance of GSCs involves an active epigenetic program with specific sets of genes that are either activated or repressed [2,22]. First, from the GSE46014 data [22], we identified gene sets that were transcriptionally activated (promoters with H3K4me3 mark) or repressed (promoters with H3K27me and with or without H3K4me3 marks) in GSCs in comparison to either normal neural stem cells (NSCs) or DGCs (Table S4). Gene set enrichment analysis (GSEA) revealed a significant depletion of GSC-specific activated gene sets compared to NSC (Figure 2C, left panel) and DGC (Figure 2D, left panel) in the METTL3-regulated transcriptome. Indeed, we found that 89.4% and 91.8% of the genes of both gene sets were downregulated in MGG8 GSCs upon METTL3 silencing (Figure 2C,D, right panels). In contrast to these observations, the GSC-specific repressed gene sets failed to show significant enrichment in the METTL3-regulated transcriptome (data not shown). These findings were further validated by the depletion of another GSC-specific activated gene set (with H3K27Ac mark) (Table S4) from GSE54047 [2] in a METTL3-regulated transcriptome (Figure 2E). In contrast, the DGC-specific, actively transcribed genes (Table S4) failed to show a significant enrichment in the METTL3 regulated transcriptome (data not shown).

To further dissect the direct contribution of m^6^A modification in this process, we analyzed a subset of GSC-specific activated gene sets that are direct targets of METTL3. We found that they showed a significant depletion in the METTL3-regulated transcriptome (Figure S2). Interestingly, the remaining set of genes from all three GSC-specific activated gene sets also showed a significant negative enrichment in the METTL3-regulated transcriptome (Figure S3) suggesting that these genes are indirect targets of METTL3. These results suggest that METTL3 plays a vital role in sustaining the expression of epigenetically activated genes in GSCs.

Next, we focused on the importance of METTL3 on the expression of a set of 19 transcription factors (TFs) that were shown to be epigenetically activated in GSCs compared to DGCs [2]. While we found most of GSC-specific transcription factors were downregulated in the METTL3-silenced condition, SOX2 alone was intensely m^6^A-modified in a METTL3-dependent manner (Figure 2F,G). The change in m^6^A modification and the transcript level of four glioma reprogramming factors (SOX2, SALL2, OLIG2 and POU3F2) and selected transcripts were validated by m^6^A RIP-PCR and RT-qPCR (Figure 2H,I). These results collectively demonstrate that the majority of the GSC-specific, actively transcribed genes are downregulated in METTL3 silenced condition, indicating the indispensable role of METTL3 and the associated m^6^A modification in sustaining the expression of epigenetically activated genes in GSCs.

### 3.4. m^6^A Modification Regulates RNA Editing

Next, we investigated the propensity of RNA editing events and the editing ratio using total RNA-Seq data of shNT-MGG8 and shMETTL3-MGG8 GSCs. At an editing ratio of ≥0.2, we found a total of 1071 events in shNT-MGG8, which was reduced by 37% in shMETTL3-MGG8 GSCs (Figure S4A). We found that most RNA editing events occurred in Alu repeat regions (72%), which were most affected by METTL3 silencing (56%) (Figure S4A). We also found that the majority of RNA editing events occurred in intronic and intergenic regions of the genome compared to exonic regions including 5′ and 3′UTRs (Figure S4B). While the overall RNA editing reduced by 37% in the METTL3-silenced condition, the events in exonic regions including 5′UTR and 3′UTR were found to be drastically reduced in METTL3-silenced GSCs (Figure S4C). Similarly, the RNA editing events, which resulted in non-synonymous and synonymous alterations, were completely abolished in METTL3-silenced GSCs (Figure S4D).

**Figure 3:**
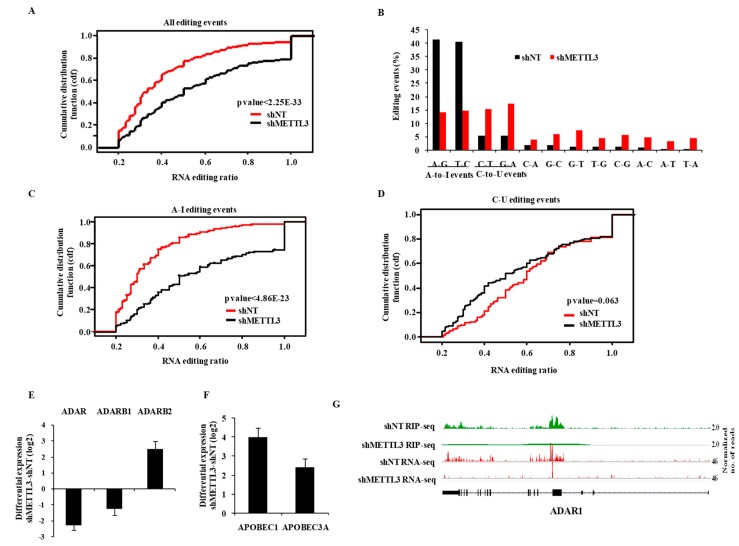
METTL3 regulates the RNA editing process. (**A**) A cumulative fraction plot of ratios for all RNA editing events in shNT vs. shMETTL3 conditions. METTL3-silencing reduced the events at varying editing ratios. (**B**) Percentage of different types of editing events between shNT and shMETTL3 cells presented as a bar diagram. A–G and C–G conversion (A-to-I editing) events decreased, while C–U and G–A conversion (C-to-U editing) events increased with METTL3-silencing. (**C**,**D**) Cumulative fraction plots of the ratio of A-to-I and C-to-U RNA editing events in shNT vs. shMETTL3 conditions are shown, respectively. METTL3-silencing reduced the A-to-I events at different editing ratios. (**E**) Differential log_2_ expression (shMETTL3-shNT) of A-to-I editing enzymes ADAR, ADARB1 and ADARB2 presented as a bar diagram. (**F**) Differential log_2_ expression (shMETTL3-shNT) of C-to-U editing enzymes APOBEC1 and APOBEC3Apresented as a bar diagram. (**G**) The genomic track of ADAR is depicted in shNT and shMETTL3-GSCs. Normalized sequence coverage of m^6^A RIP peaks and RNA-Seq reads is indicated above the gene architecture in UCSC format. Thin black boxes represent the 5′ and 3′UTRs, thick black boxes represent the coding sequences, and thin lines represent introns. ADAR showed m^6^A RIP peaks in control GSCs, which was depleted in shMETTL3 GSCs.

The reduced occurrence of RNA editing events in the shMETTL3 condition was further confirmed by the significant reduction of the cumulative fraction of RNA editing events in shMETTL3-MGG8 GSCs at varying RNA editing ratios (Figure 3A). The majority of editing events were found to be A-to-I and C-to-U types (Figure S4E). Interestingly, we noticed a significant reduction in A-to-I events, but a significant increase in C-to-U events (Figure 3B; compare red bars to black bars). This was also confirmed by cumulative RNA editing events in shMETTL3-GSCs at varying RNA editing ratios (Figure 3C,D). Next, we found that there was no significant overlap between RNA edited bases and m^6^A peaks in shNT-GSCs (data not shown) suggesting that the regulation of RNA editing by METTL3 might be independent of m^6^A modification function. Further, we found that both ADAR and ADARB1, which encode the functional A-to-I editing enzymes, were downregulated, while APOBEC1 and APOBEC3A, the key enzymes of C-to-U editing, were significantly upregulated in shMETTL3-GSCs (Figure 3E,F). The inhibitory enzyme for A-to-I editing (i.e., ADARB2) [23] was upregulated in the METTL3-silenced condition. Interestingly, we also found that ADAR was a direct target of METTL3 (Figure 3G; Tables S1 and S2). Collectively, these results demonstrate that METLL3 regulates the RNA editing, particularly in exonic regions by altering the levels of RNA editing enzymes.

### 3.5. Functional Association of RNA Processing Factors with m^6^A Modification

**Figure 4:**
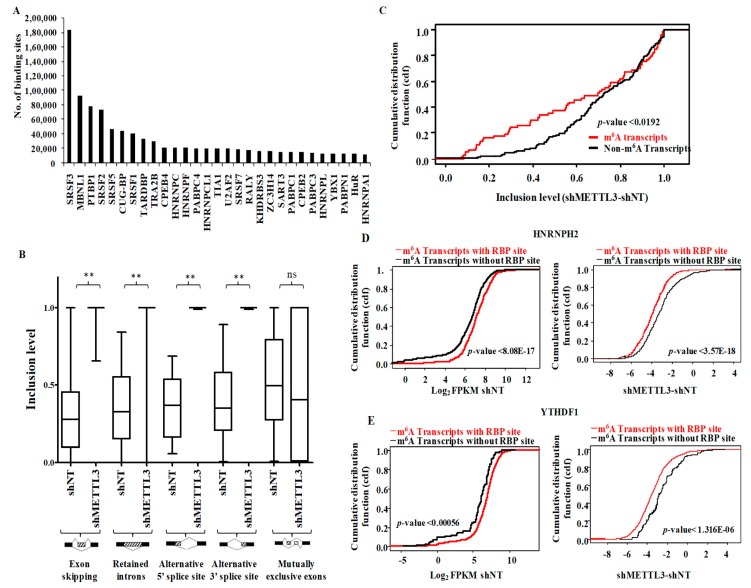
Inter-play of METTL3-dependent m^6^A modification in post-transcriptional RNA processing. (**A**) The Top 30 RNA binding proteins (RBPs) that bind to m^6^A modified regions. The number of consensus sites present within m^6^A peaks for each RBP is plotted according to the RBP map tool. (**B**) Inclusion levels for five types (exon skipping, intron retention, 3′ASS, 5′ASS and mutually exclusive exons) of splicing events in shNT-GSCs and shMETTL3-GSCs were plotted. Exon skipping, intron retention, 3′ASS and 5′ASS showed significant reduction in shMETTL3-GSCs compared to shNT-GSCs. *p*-values were calculated by Wilcoxon test. (**C**) Cumulative fraction plot of differential inclusion levels for exon skipping process in m^6^A-modified genes compared to non-m^6^A genes. (**D**) Genes with m^6^A peaks encompassing the consensus sequence of RNA binding protein HNRNPH2 were compared with m^6^A-modified genes, which lack these RBP sites. The distribution of abundance (FPKM) in shNT-GSCs and differential regulation post-METTL3 silencing were plotted independently as cumulative fraction. (**E**) Genes with m^6^A peaks encompassing the consensus sequence of RNA binding protein YTHDF1 were compared with m^6^A-modified genes, which lack these RBP sites. The distribution of abundance (FPKM) in shNT-GSCs and differential regulation post-METTL3 silencing were plotted independently as cumulative fraction.

Examination of the presence of RBP binding motifs across the m^6^A peaks obtained from shNT-MGG8 GSCs revealed the presence of binding sites in the m^6^A peak areas for 94 RBPs. Among the top m^6^A-RBP pairs, we observed a high enrichment of motifs of several SRSF family proteins, which are involved in mRNA splicing and export (Figure 4A). Gene ontology (GO) analysis of the top-most enriched RBPs revealed an overrepresentation of terms related to RNA splicing and transport (Figure S5A). The above observation instigated an examination of the regulation of alternative splicing events by METTL3. An analysis of differential splicing events between the shNT-MGG8 and shMETTL3-MGG8 GSC transcriptome, revealed a significant aberrant accumulation of exon inclusion, retained introns, along with an emergence of 5′ASS sites and novel 3′ASS events in METTL3-silenced cells (Table S5 and Figure 4B). The pathway enrichment analysis for each type of aberrant alternative splicing category produced terms as mRNA splicing, TP53 degradation, VEGF signaling, translation initiation, NMD machinery, cell cycle, telomere synthesis and nucleotide extension repair (Figure S5B). These results suggest that METTL3 silencing deregulates these pathways by producing distinct isoforms with deleted/added domains.

Next, we examined the involvement of METTL3-dependent m^6^A modification in aberrant splicing processes. The cumulative distribution fraction showed the high inclusion level seen in METTL3-silenced condition for m^6^A modified transcripts, suggesting that the exon skipping events were dependent on m^6^A modification (Figure 4C). Interestingly, the skipped exon regions encompassed m^6^A-modified locations in shNT-GSCs, suggesting that METTL3-dependent modification is essential for exon skipping (Figure S5C). However, none of the other types of alternative splicing investigated here showed dependency on m^6^A modification, implicating a methylation-independent function of METTL3 in regulating these splicing events (Table S5).

As an example, we show the splice variants of MDM2 (Figure S6), which skipped exons 3, 4, 5 or 9 (MDM2-1 to MDM2-4) in METTL3 silenced GSCs. Exons 3, 4 and 5 span the p53 interaction domain, whereas exon 9 is part of the nuclear export/localization signal. In control MGG8 GSCs, we detected these isoforms with skipped exons, which were significantly reduced in METTL3-silenced cells suggesting a key role for METTL3 in exon skipping of MDM2 (Table S5). In agreement, we indeed observed increased early and late apoptosis of METTL3-silenced cells [7]. Further studies are warranted to delineate the functional relevance of skipped exons and METTL3 in GSCs.

Next, we investigated the functional coordination between RBPs (*n* = 48) that are m^6^A readers [24] or involved in RNA decay pathways [25] with m^6^A. The cumulative distribution function of the differential transcript levels (shMETTL3-shNT) after METTL3 silencing between m^6^A-modified transcripts with and without RBP bindings sites revealed three categories of RBPs: m^6^A stabilizers, m^6^A destabilizers and non-m^6^A regulators (Table S6). We found that many RBPs (20 out of 48; 41.6%) that had overlapping RBP binding sites with m^6^A peaks enhanced the abundance of the transcripts in a METTL3-dependent manner as its silencing reduced the transcript stability. Of these, two proteins—HNRNPH2 and YTHDF1—had maximum stabilizing impact on m^6^A-modified transcripts (Figure 4D,E). This implies that these RBPs play a collaborative RNA-stabilizing role along with the m^6^A modification. Hence, they may act as putative m^6^A readers that enhance the stability of the modified targets. Earlier reports suggest that HNRNPH2 regulates the polyadenylation process and preserves the transcripts from degradation [26]. HNRNPU binds to the 3′UTR of several transcripts, including TNFα, and stabilizes the mRNA [27]. Taken together, these findings demonstrate that METTL3 collaborates with RNA splicing factors and other RBPs in controlling RNA splicing and stability.

### 3.6. Regulation of Long Non-Coding RNAs and miRNAs by m^6^A-Methylome

Additional analysis of RNA-Seq data revealed that a considerable fraction of non-coding RNAs are also regulated by METTL3-mediated m^6^A modification (Figure S7A,B and Table S3). In contrast to protein-coding genes, the majority of the regulated lincRNAs were upregulated in METTL3-silenced MGG8 GSCs (Figure S7B). Further investigation found that lincRNAs were less abundant compared to protein-coding genes (Figure S7C; compare black line with red line), in agreement with previous reports [28]. However, m^6^A-modified transcripts of both protein-coding and lincRNAs had higher abundance and were lost upon METTL3 silencing (Figure S7D,E; compare red line with black line). The loss of m^6^A peaks and transcript downregulation for XIST, MALAT1, and H19 lincRNA are shown in Figure S7F. XIST and MALAT1 are previously well-studied m^6^Atargets in different cell types, suggesting a functional conservation of this modification [29,30]. Collectively, these results establish that METTL3-mediated m^6^A modification is essential for RNA stabilization, regardless of whether it is of the coding or non-coding type. However, the reason behind the upregulation of the majority of lincRNAs in the METTL3-silenced condition remains to be investigated. It is interesting to note that only a small fraction of lincRNAs (0.25%) were m^6^A modified as against protein-coding transcripts (6.04%).

The analysis also revealed a differential regulation of 22 miRNAs (1.4% of detected miRNAs) in METTL3-silenced MGG GSCs (Figure S7G and Table S3). In addition to the direct regulation of miRNAs by METTL3, we identified a network of enriched, experimentally validated miRNA/mRNA pairs in which target transcripts were intensely modified by METTL3 at the 3′UTR and the target gene required METTL3 for stable expression (Table S7). We identified VEGFA, SEPT2 and IGF1R as top-ranking genes that are targeted by many miRNAs at the 3′UTR m^6^A-modified sites (Figure S7H). These genes mediate invasion, migration, and angiogenesis function in glioblastoma and other cancers [31,32,33]. The miRNAs miR-16, miR-15a, miR-15b and miR-100, which target these genes, were upregulated in our cohort of miRNA profiling of glioblastoma tissues compared to control brain (Table S7 and Figure S7H) [34]. These observations suggest that m^6^A modification at their 3′UTR may rescue these transcripts from degradation by miRNAs even though the miRNAs are upregulated in glioblastoma. However, given the absence of overlap between modified m^6^A consensus sites and miRNA binding sites (data not shown), we propose that m^6^A modification of 3′UTR may create a unique conformation that may prevent miRNA binding to the target transcripts. Further studies are required to substantiate the outcome of the miRNA interaction with m^6^A modified target mRNAs.

### 3.7. Integrated Pathway Analysis of Regulated Transcriptome Implies an Oncogenic Role for METTL3

**Figure 5:**
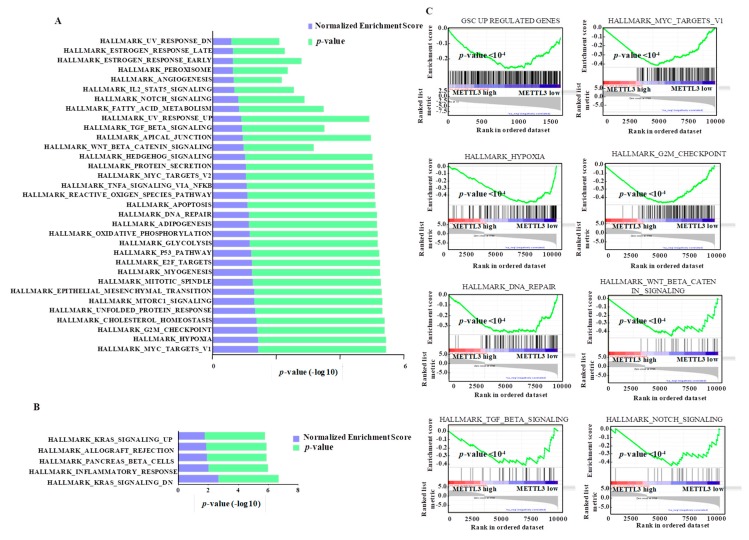
Pathways regulated by METTL3-silencing in GSCs. Gene set enrichment analysis (GSEA) was performed for genes that are regulated by METTL3 at the transcript-level with more than two-fold regulation (*p* < 0.05). Hallmark pathways depleted (**A**) and enriched (**B**) in METTL3-silenced GSCs are depicted. NES (normalized enrichment score) and *p*-values are given as a bar graph. (**C**) For selected gene sets, gene set enrichment plots depicting negative regulation with significant *p*-value (<0.05) after METTL3 silencing are shown.

To understand the crucial role played by METTL3 in glioma development and progression, an unbiased pathway analysis using gene sets that correspond to various hallmarks of defined biological states in the METTL3-regulated transcriptome was carried out. GSEA revealed a significant depletion of a large number of gene sets (*n* = 33), while there were only five positively enriched ones (Figure 5A,B). It is particularly noteworthy that most negatively enriched pathways were oncogenic pathways such as MYC, mTORC1, E2F, TGF-β, NOTCH, TNFα/NFKB signaling, hedgehog signaling, and Wnt β-catenin signaling (Figure 5C). In addition, important cell cycle and DNA repair pathways (i.e., G2/M checkpoint, DNA repair and mitotic spindle) were found negatively enriched (Figure 5A). The list also included other key metabolic pathways related to transformation, such as hypoxia, unfolded protein response, oxidative phosphorylation, glycolysis, reactive oxygen species pathway, and fatty acid metabolism (Figure 5A).

Next, we carried out a gene ontology analysis to differentiate the functions of direct and indirect METTL3 targets. Panther pathway enrichment essentially identified a non-overlapping set of pathways for direct versus indirect targets of METTL3 (Table S8). While the direct targets of METTL3 were enriched for major oncogenic pathways such as NOTCH, VEGF, angiogenesis, glycolysis and hedgehog signaling, the indirect targets were enriched in the RAS pathway, MAPK pathway, GPCR, cadherin signaling pathway and cell cycle (Figure S8A,B). Interestingly, two pathways (i.e., EGFR and integrin signaling) are regulated by both direct and indirect targets of METTL3.

**Figure 6:**
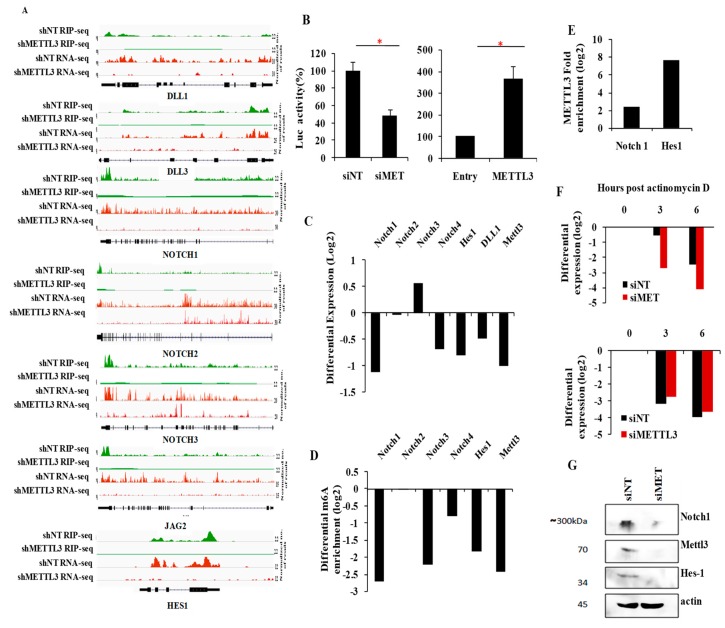
METTL3-dependent m^6^A modification positively regulates NOTCH signaling. (**A**) The genomic tracks for Notch pathway genes–DLL1, DLL3, NOTCH1, NOTCH2, NOTCH3, JAG2 and HES1 are depicted in both shNT vs. shMETTL3 m^6^A RIP-Seq and RNA-Seq. Normalized sequence coverage of m^6^A RIP peaks and RNA-Seq reads is indicated above the gene architecture in UCSC format. Thin black boxes represent the 5′ and 3′UTRs and thick black boxes represent the coding sequences and thin lines represent introns. (**B**) HES-1 promoter luciferase activity was measured after silencing (left panel) and over expression of METTL3 (right panel). Luciferase activity was plotted as percentage after normalization to the control cells. Silencing of METTL3 reduced NOTCH activation while over-expression induced the NOTCH pathway. (**C**) Transcript levels of NOTCH pathway members, which have multiple METTL3-dependent m^6^A RIP-Seq peaks, were measured post-METTL3silencing. Differential expression was measured compared to siNT condition by real-time qPCR and plotted as log_2_ ratio. (**D**) Differential m^6^A enrichment of NOTCH 1, NOTCH 2, NOTCH 3, NOTCH 4 and HES1 genes were measured by m^6^A RIP-PCR after METTL3-silencing and were plotted as log_2_ ratio. The enrichment was normalized to siNT condition, (**E**) Enrichment of NOTCH1 and HES1 genes were measured by METTL3 RIP-PCR and were plotted as log_2_. Anti-METTL3 immunoprecipitation (IP) was normalized to IgG control IP. (**F**) NOTCH1 (top) and HES1 (bottom) transcripts were measured at indicated time points post actinomycin D (5 μg/mL) treatment in siNT and siMETTL3 condition by reverse transcription-qPCR. The log_2_ ratio of remaining NOTCH1 was plotted using linear regression after normalizing to the 0th hour of respective condition. (**G**) Protein levels of full-length NOTCH1 and Hes1 after METTL3 knockdown. The *p*-value was analyzed by Student’s *t*-test. *** *p*-value <0.001, ** *p*-value <0.01, * *p*-value <0.05. Error bars represent standard deviation.

To further validate these findings, we investigated the importance of METTL3 in NOTCH pathway activation. GSEA also demonstrated a significant depletion of another Notch target gene set [35] (Figure S9A). Several notch pathway genes (i.e., notch ligands DLL1, DLL3 and JAG2, notch receptors NOTCH1, NOTCH2 and NOTCH3, and the key notch target HES1) had METTL3-dependent m^6^A peaks, and their transcript levels were downregulated in METTL3-silenced cells (Figure 6A and Figure S9B). We also demonstrated that NOTCH pathway activation requires METTL3 as seen by the significant reduction in luciferase activity from NOTCH-dependent HES1 promoter reporter after METTL3 silencing (Figure 6B, left panel). In addition, the notch pathway-dependent luciferase activity was induced in glioma cells upon exogenous overexpression of METTL3 (Figure 6B, right panel). Next, we demonstrated that transcript levels of NOTCH pathway members NOTCH1, NOTCH4, DLL1 and HES-1, which carry METTL3-dependent m^6^A peaks, were downregulated in METTL3-silenced glioma cells (Figure 6C, Table 1). To further confirm the METTL3-mediated m^6^A modification of these transcripts, we performed m^6^A RIP PCR post METTL3 silencing. We found that NOTCH1, NOTCH3, NOTCH4, and HES1 showed significant reduction of m^6^A modification in siMETTL3 cells (Figure 6D, Table 1). As NOTCH1 and HES1 were strongly downregulated in METTL3-silenced cells and their transcripts carry a METTL3-dependent m^6^A modification, as shown above, we quantified the METTL3 interaction by METTL3 RIP-PCR. Both transcripts showed positive enrichment confirming the METTL3 binding to these mRNAs (Figure 6E). We also demonstrated the RNA stabilization of NOTCH1 transcript by METTL3 by actinomycin D-RNA stability assay post METTL3 silencing (Figure 6F, top) while the HES1 transcript level remained unchanged (Figure 6F, bottom). In accordance with previous results, METTL3 silencing resulted in downregulation of NOTCH1 and HES1 protein expression (Figure 6G). Collectively, from these findings, we conclude that the METTL3 enhances the activity of the Notch pathway in glioma. Further, we conclude that successful execution of oncogenic pathways requires METTL3 and the associated m^6^A modification, thus implicating an oncogenic role for METTL3 through its direct and indirect targets in glioma.

## 4. Discussion

The gene expression undergoes transient and rapid changes by m^6^A modification. Although m^6^A and the related enzymes METTL3 and FTO are reported to be essential for neuronal functions [36,37], the role of METTL3-mediated m^6^A modification in gliomagenesis remains obscure. Here, we define a global METTL3-mediated m^6^A epitranscriptome roadmap of GSCs. m^6^A modification is catalyzed by methyl transferases METTL3 and METTL14. Although initial reports suggest a redundant function for these enzymes [4], structural studies decode that only METTL3 carries the catalytic domain of SAM-binding, while METTL14 acts as an augmenting subunit [8]. Accordingly, our study demonstrates that silencing METTL3 depleted the majority of m^6^A peaks, suggesting that METTL3 is the key methylase in GSCs. METTL3-mediated m^6^A modification regulates three rate limiting factors of RNA metabolism: splicing [38,39], RNA stability [7,40,41], and translation [42]. We found that transcripts with m^6^A modification showed high abundance and the majority of the targets were downregulated post-depletion of METTL3. Further, we show that m^6^A modification requires a specific conformation besides the presence of a binding site as shown previously by our laboratory [7]. We conclude that METTL3 and the associated m^6^A modification promote RNA stabilization, thus playing a crucial role in the expression of highly abundant transcripts. Previous reports by Cui et al. and Zhang et al. suggested that METTL3-dependent m^6^A modification marks the cells for differentiation and inhibits glioma stem cell growth [43,44]. Cui et al. showed that METTL3 inhibition increases ADAM19 transcript and enhances glioma cell proliferation. Further, m^6^A modification stabilizes FOXM1 matured mRNA and enhances differentiation [44]. The variation in the observations from our report can be attributed to the differences in culture expansion condition and inter-tumor heterogeneity of GBMs. In addition, the modification shows context-dependent functions, since the abundance of readers and anti-readers that interpret m^6^A and the compartment-dependent functions of these readers govern the outcome [24]. In agreement with our study, the m^6^A adaptor protein WTAP enhances the proliferation, invasion, and migration in glioma cells which suggests the contextual function of m^6^A modification [45]. Additionally, various studies in different cancer systems supported that METTL3 and m^6^A modification are involved in the transformation and tumor progression [7,29,42,46,47,48,49,50,51,52,53].

For the first time, we reveal that the expression of genes that are poised for transcription with active histone marks is augmented by METTL3-mediated stabilization of transcripts. METTL3-mediated m^6^A modification works cooperatively with active histone marks and transcription factors to sustain the high expression of genes that are essential for GSC maintenance. Our analysis showed that both direct and indirect targets of METTL3 take part in the function. The GSC-specific genes, which carry repressive marks, were not influenced by METTL3-dependent m^6^A modification. We speculate that METTL3 activity and a histone-dependent transcriptional switch might work in a positive feedback loop to maintain the homeostasis of a cell. Additional studies are required to investigate the putative mechanism behind the coordination between histone-mediated gene activation and the m^6^A epitranscriptome.

Our analysis showed that METTL3 regulates A-to-I and C-to-U editing. The majority of the editing events laid within Alu regions while a significant number altered the coding regions of the transcripts. We failed to observe any overlap between m^6^A-modified consensus sites with RNA editing sites. However, the results cannot rule out the fact that the secondary structural alterations induced by the modification are mediating the editing event [7,54,55]. According to our results, the effect of METTL3 on editing is indirect, since the editing enzyme ADAR was downregulated while APOBEC was upregulated in METTL3-silenced GSCs, which correlates with the differential editing ratio of A-to-I and C-to-U events in shMETTL3 GSCs.

We identified a comprehensive interactive map of putative m^6^A readers from the m^6^A-modified sites and their coordination functions in splicing and in augmenting or preventing RNA decay. The conformational alterations induced by m^6^A modification may specifically recruit these readers to the site, which may further help in the processing of mRNAs. The majority of the m^6^A-modified sites overlap with splicing factor binding sites. In agreement with a previous report [38], we observed aberrant splicing, including elevated intron retention and exon inclusion in METTL3-silenced cells. In addition to splicing factor recruitment, m^6^A may facilitate the structural reorganization required for catalysis of splice ends. Exclusively, the modification correlates with the exon skipping function while other functions (e.g., intron detention, 3′ASS, and 5′ASS) remain unaffected by the presence of m^6^A modification at the site. Since unproductive splicing events of core spliceosome genes lead to early stop codons or nonsense-mediated decay [56], we speculate that METTL3 may additionally maintain the homeostasis of the functional form of splicing factors. Xiao et al. [39] reported that the m^6^A reader YTHDC1 recruits SRSF3 to m^6^A-modified sites and leads to exon inclusion. In contrast, Molinie et al. identified that m^6^A-modified transcripts show a high incidence of exon skipping [38]. A recent study demonstrated that the deletion of Ime4 (paralog of METTL3 in *Drosophila*) in *Drosophila* increased the inclusion rate of male-specific exon into the Sxl gene, which is crucial for female sex determination and dosage compensation [37]. In agreement, our analysis showed that the majority of the skipped exons laid within the m^6^A-modified sites, suggesting that m^6^A modification may help in splicing repressors binding to exons. We identified the inclusion of several exons in MDM2 post-METTL3 silencing. Previous reports identified an abundance of various defective MDM2 variants that are devoid of p53 binding and NES/NLS regions in cancer cells [57]. These isoforms lack p53 binding and portray oncogenic activities independent of p53 in cancer cells [58]. Stainmanetal showed that the MDM2-bvariant induces tumor formation, activates NFκB and inhibits apoptosis in a p53-independent manner [59]. In addition, in mutant p53 cell lines, MDM2 variants enhance the tumorigenic activity mediated by mutant p53 [57].

Previous studies showed that m^6^A modification is involved in RNA stabilization [7,60] or RNA decay [40,41,61] in a context-dependent manner. We found a large number of RBP binding sites present within m^6^A-modified regions and the majority of the RBPs analyzed enhanced the RNA stability in coordination with m^6^A modification. Unlike the previous studies [41,62], the m^6^A-modified genes with binding sites for the YTH domain containing m^6^A readers YTHDF2 and YTHDF3 showed elevated expression compared to non-target m^6^A genes. Although YTHDF proteins were shown to shuttle the mRNAs to P-bodies [61,63], they promoted ribosomal loading onto modified mRNAs, suggesting that they may protect the actively translating mRNA pool from degradation [40,41]. hnRNPH2, which is known to enhance the cleavage of 3′UTR processing and maintain the stability of mRNAs, also showed a positive influence on m^6^A-modified transcripts [64].

In addition to protein-coding genes, we examined METTL3-driven regulation by m^6^A modification on lincRNAs and miRNAs of GSCs. We found that a considerable proportion of lincRNAs were altered by METTL3 silencing. In contrast to the protein-coding genes, the majority of lincRNAs were upregulated in shMETTL3-GSCs. We speculate that METTL3 might regulate the lincRNA-specific degradation machinery. However, few of the lincRNAs were downregulated post-METTL3 silencing and they carried METTL3-dependent m^6^A modifications. The results suggest that m^6^A modification is involved in a global phenomenon of RNA stabilization. Although a previous report proved that m^6^A modification is involved in primary miRNA processing [65], due to the isolation method followed, the number of detected miRNAs was few and no miRNA was identified as a direct target. Few miRNAs were regulated and the majority were downregulated by METTL3 silencing. We further attempted an interactive analysis between miRNA-mediated gene regulation and the m^6^A epitranscriptome. We identified direct downregulated targets post-METTL3 silencing which showed overlapping m^6^A peaks with miRNA seed sequences at their 3′UTRs. The microRNA seed sequence and m^6^A consensus site lie within 30 nt vicinity, suggesting that m^6^A may alter the accessibility of transcripts to miRNA binding by recruiting other RNA-binding proteins to the target sites. Although the miRNA seed sequence does not overlap with a modified consensus site, the regulation might be mediated by secondary/tertiary conformation. We identified the oncogenes VEGFA, SEPT2 and IGF1R as probable targets, which might be rescued by m^6^A modification from miRNA inhibition. Further, miRNA-15b [66] and miRNA-29b [67], which were identified in the analysis were previously reported to induce apoptosis and inhibit stem-like characteristics in glioma cells. The functional coordination between these miRNAs and the m^6^A modification in glioma is open for future studies.

Comprehensive analysis of direct and indirect targets with RNA regulation post METTL3 silencing showed that it is indispensable for key glioblastoma-related oncogenic pathways such as NOTCH, NFκB, Wnt, c-Myc, and TGF-β. Other important processes such as DNA repair, G2M check point and hypoxia are also found to be inhibited in METTL3-silenced cells. Thus, the pathways involved in glioma stem cell maintenance and tumorigenesis are positively influenced by METTL3-mediated RNA stabilization implying an oncogenic role for METTL3 in GSCs. In accordance with our previous report [7], we found glioma stem cell-specific genes were downregulated post-METTL3 inhibition. Further, we also provide evidence that the NOTCH pathway activation requires METTL3. Our results suggest that METTL3 modifies and enhances the expression of several NOTCH pathway members. NOTCH1 and HES1 are direct targets of METTL3.

## 5. Conclusions

Methyltransferase-like 3 (METTL3) -dependent m^6^A-RIP Seq combined with transcriptome analysis paved the way for target identification and helped in correlating the RNA methylome to various biological processes. Our study uncovered novel RNA metabolic events that depend on METTL3 and the resultant m^6^A modification. METTL3-mediated m^6^A modification coordinates in maintaining the GSC-specific gene expression along with the histone open complex. METTL3 silencing deregulates RNA editing and splicing. Our analysis identified putative m^6^A readers and their function in stabilizing/destabilizing modified transcripts. Overall, this study implicates an indispensable role for METTL3 in the active execution of oncogenic pathways, which are important for GSC maintenance.

## Figures and Tables

**Table 1:** List of the real-time primers used.

Gene Name	Primers (5′-3′)
METTL3-FP	ACCTATGCTGACCATTACCAAG
METTL3-RP	CTGTTGGTTCAGAAGGCTCTC
SOX2-FP	AACCCCAAGATGCACAACTC
SOX2-RP	GCTTAGCCTCGTCGATGAAC
OLIG2-FP	CCAGAGCCCGATGACCTTTT
OLIG2-RP	AGGACGACTTGAAGCCACTG
POU3F2-FP	TGACGATCTCCACGCAGTAG
POU3F2-RP	GGCAGAAAGCTGTCCAAGTC
SALL2-FP	TAATCTCGGACTGCGAAGGT
SALL2-RP	TAGAACATGCGTTCTGGTGG
ATP5G-FP	CCAGACGGGAGTTCCAGAC
ATP5G-RP	GACGGGTTCCTGGCATAGC
DLL1-FP	GCAGCTCTTCACCCTGTTCT
DLL1-RP	GGTGCAGGAGAAGTCGTTCA
NOTCH1-FP	GAGGCCTGGCAGACTATGC
NOTCH1-RP	CTTGTACTCCGTCAGCGTGA
HES1-FP	AGTGAAGCACCTCCGGAAC
HES1-RP	TCACCTCGTTCATGCACTC
NOTCH2-fp	CTGTGAGTGTCTGAAGGGTTATG
NOTCH2-rp	GGCACTGGAAACGATTGACTTT
NOTCH3-fp	CGTGGCTTCTTTCTACTGTGC
NOTCH3-rp	CGTTCACCGGATTTGTGTCAC
NOTCH4-fp	CTGGGTGTCAATGGAGAGGGA
NOTCH4-rp	GGGTGAGACGTGCCAGTTTC

## Supplementary Materials

The following are available online at http://www.mdpi.com/2073-4425/10/2/141/s1, Figure S1: m^6^A peak distribution, Figure S2: GSC specific histone activated genes relate to METTL3 direct targets, Figure S3: GSC specific histone activated genes relate to METTL3 indirect targets, Figure S4: Distribution of METTL3 dependent RNA editing events, Figure S5: METTL3 and m^6^A modification in RNA processing, Figure S6: METTL3 regulate the exon skipping of MDM2, Figure S7: Regulation of lincRNAs and miRNAs by METTL3 in GSCs, Figure S8: Pathways regulated by direct and indirect METTL3 targets, Figure S9: Regulation of NOTCH pathway by METTL3 mediated m^6^A modification, Table S1: m^6^A peaks identified in shNT GSC, Table S2: m^6^A peaks identified in shMETTL3 GSC, Table S3: Differentially expressed Genes in shMETTL3 GSC - shNT GSC, Table S4: GSC specific gene sets used for analysis, Table S5: Differential splicing events between shNT and shMETTL3 GSC, Table S6: Coordination of m^6^A with RBPs, Table S7: miRNAs binding to m^6^A modified 3’UTR genes, Table S8: DAVID pathway analysis for direct and indirect targets of METTL3.

## List of Abbreviations

m^6^A	*N*^6^-methyladenosine
METTL3	methyltransferase-like3
METTL14	methyl transferase-like 14
FTO	fat mass and obesity-associated protein
ALKBH5	alkB homolog 5
GSC	glioma stem-like cell
DGC	differentiated glioma cell
NSC	normal neural stem cell

## References

[1] Wang J., Ma Y., Cooper M.K. (2013). Cancer stem cells in glioma: Challenges and opportunities. Transl. Cancer Res..

[2] Suva M.L., Rheinbay E., Gillespie S.M., Patel A.P., Wakimoto H., Rabkin S.D., Riggi N., Chi A.S., Cahill D.P., Nahed B.V. (2014). Reconstructing and reprogramming the tumor-propagating potential of glioblastoma stem-like cells. Cell.

[3] Auffinger B., Tobias A.L., Han Y., Lee G., Guo D., Dey M., Lesniak M.S., Ahmed A.U. (2014). Conversion of differentiated cancer cells into cancer stem-like cells in a glioblastoma model after primary chemotherapy. Cell Death Differ..

[4] Liu J., Yue Y., Han D., Wang X., Fu Y., Zhang L., Jia G., Yu M., Lu Z., Deng X. (2014). A METTL3-METTL14 complex mediates mammalian nuclear RNA N^6^-adenosine methylation. Nat. Chem. Biol..

[5] Zheng G., Dahl J.A., Niu Y., Fedorcsak P., Huang C.M., Li C.J., Vagbo C.B., Shi Y., Wang W.L., Song S.H. (2013). ALKBH5 is a mammalian RNA demethylase that impacts RNA metabolism and mouse fertility. Mol. Cell.

[6] Jia G., Fu Y., Zhao X., Dai Q., Zheng G., Yang Y., Yi C., Lindahl T., Pan T., Yang Y.G. (2011). *N*^6^-methyladenosine in nuclear RNA is a major substrate of the obesity-associated FTO. Nat. Chem. Biol..

[7] Visvanathan A., Patil V., Arora A., Hegde A.S., Arivazhagan A., Santosh V., Somasundaram K. (2018). Essential role of METTL3-mediated m^6^A modification in glioma stem-like cells maintenance and radioresistance. Oncogene.

[8] Wang P., Doxtader K.A., Nam Y. (2016). Structural Basis for Cooperative Function of Mettl3 and Mettl14 Methyltransferases. Mol. Cell.

[9] Ramaswami G., Zhang R., Piskol R., Keegan L.P., Deng P., O’Connell M.A., Li J.B. (2013). Identifying RNA editing sites using RNA sequencing data alone. Nat. Methods.

[10] Li H., Durbin R. (2009). Fast and accurate short read alignment with Burrows-Wheeler transform. Bioinformatics.

[11] McKenna A., Hanna M., Banks E., Sivachenko A., Cibulskis K., Kernytsky A., Garimella K., Altshuler D., Gabriel S., Daly M. (2010). The Genome Analysis Toolkit: A MapReduce framework for analyzing next-generation DNA sequencing data. Genome Res..

[12] Shen S., Park J.W., Lu Z.X., Lin L., Henry M.D., Wu Y.N., Zhou Q., Xing Y. (2014). rMATS: Robust and flexible detection of differential alternative splicing from replicate RNA-Seq data. Proc. Natl. Acad. Sci. USA.

[13] Dobin A., Davis C.A., Schlesinger F., Drenkow J., Zaleski C., Jha S., Batut P., Chaisson M., Gingeras T.R. (2013). STAR: Ultrafast universal RNA-seq aligner. Bioinformatics.

[14] Kim D., Pertea G., Trapnell C., Pimentel H., Kelley R., Salzberg S.L. (2013). TopHat2: Accurate alignment of transcriptomes in the presence of insertions, deletions and gene fusions. Genome Biol..

[15] Love M.I., Huber W., Anders S. (2014). Moderated estimation of fold change and dispersion for RNA-seq data with DESeq2. Genome Biol..

[16] Subramanian A., Tamayo P., Mootha V.K., Mukherjee S., Ebert B.L., Gillette M.A., Paulovich A., Pomeroy S.L., Golub T.R., Lander E.S. (2005). Gene set enrichment analysis: A knowledge-based approach for interpreting genome-wide expression profiles. Proc. Natl. Acad. Sci. USA.

[17] Huang da W., Sherman B.T., Lempicki R.A. (2009). Systematic and integrative analysis of large gene lists using DAVID bioinformatics resources. Nat. Protocols.

[18] Paz I., Kosti I., Ares M., Cline M., Mandel-Gutfreund Y. (2014). RBPmap: A web server for mapping binding sites of RNA-binding proteins. Nucleic Acids Res..

[19] Agarwal V., Bell G.W., Nam J.-W., Bartel D.P. (2015). Predicting effective microRNA target sites in mammalian mRNAs. eLife.

[20] Shannon P., Markiel A., Ozier O., Baliga N.S., Wang J.T., Ramage D., Amin N., Schwikowski B., Ideker T. (2003). Cytoscape: A software environment for integrated models of biomolecular interaction networks. Genome Res..

[21] Bailey T.L., Boden M., Buske F.A., Frith M., Grant C.E., Clementi L., Ren J., Li W.W., Noble W.S. (2009). MEME SUITE: Tools for motif discovery and searching. Nucleic Acids Res..

[22] Rheinbay E., Suva M.L., Gillespie S.M., Wakimoto H., Patel A.P., Shahid M., Oksuz O., Rabkin S.D., Martuza R.L., Rivera M.N. (2013). An aberrant transcription factor network essential for Wnt signaling and stem cell maintenance in glioblastoma. Cell Rep..

[23] Oakes E., Anderson A., Cohen-Gadol A., Hundley H.A. (2017). Adenosine deaminase that acts on RNA 3 (ADAR3) binding to glutamate receptor subunit B pre-mRNA inhibits RNA editing in glioblastoma. J. Biol. Chem..

[24] Visvanathan A., Somasundaram K. (2018). mRNA Traffic Control Reviewed: *N*^6^-Methyladenosine (m^6^A) Takes the Driver’s Seat. BioEssays.

[25] Van Nostrand E.L., Freese P., Pratt G.A., Wang X., Wei X., Blue S.M., Dominguez D., Cody N.A.L., Olson S., Sundararaman B. (2018). A Large-scale binding and functional map of human RNA binding proteins. bioRxiv.

[26] Alkan S.A., Martincic K., Milcarek C. (2006). The hnRNPs F and H2 bind to similar sequences to influence gene expression. Biochem. J..

[27] Yugami M., Kabe Y., Yamaguchi Y., Wada T., Handa H. (2007). hnRNP-U enhances the expression of specific genes by stabilizing mRNA. FEBS Lett..

[28] Kristensen V.N., Lingjaerde O.C., Russnes H.G., Vollan H.K., Frigessi A., Borresen-Dale A.L. (2014). Principles and methods of integrative genomic analyses in cancer. Nat. Rev. Cancer.

[29] Liu N., Dai Q., Zheng G., He C., Parisien M., Pan T. (2015). *N*^6^-methyladenosine-dependent RNA structural switches regulate RNA-protein interactions. Nature.

[30] Patil D.P., Chen C.K., Pickering B.F., Chow A., Jackson C., Guttman M., Jaffrey S.R. (2016). m^6^A RNA methylation promotes XIST-mediated transcriptional repression. Nature.

[31] Zhou Y.H., Tan F., Hess K.R., Yung W.K. (2003). The expression of PAX6, PTEN, vascular endothelial growth factor, and epidermal growth factor receptor in gliomas: Relationship to tumor grade and survival. Clin. Cancer Res..

[32] Kim D.S., Hubbard S.L., Peraud A., Salhia B., Sakai K., Rutka J.T. (2004). Analysis of mammalian septin expression in human malignant brain tumors. Neoplasia.

[33] Zhang N., Liu L., Fan N., Zhang Q., Wang W., Zheng M., Ma L., Li Y., Shi L. (2016). The requirement of SEPT2 and SEPT7 for migration and invasion in human breast cancer via MEK/ERK activation. Oncotarget.

[34] Rao S.A., Santosh V., Somasundaram K. (2010). Genome-wide expression profiling identifies deregulated miRNAs in malignant astrocytoma. Mod. Pathol..

[35] Nguyen B.C., Lefort K., Mandinova A., Antonini D., Devgan V., Della Gatta G., Koster M.I., Zhang Z., Wang J., Tommasi di Vignano A. (2006). Cross-regulation between Notch and p63 in keratinocyte commitment to differentiation. Genes Dev..

[36] Hess M.E., Hess S., Meyer K.D., Verhagen L.A., Koch L., Bronneke H.S., Dietrich M.O., Jordan S.D., Saletore Y., Elemento O. (2013). The fat mass and obesity associated gene (*Fto*) regulates activity of the dopaminergic midbrain circuitry. Nat. Neurosci..

[37] Lence T., Akhtar J., Bayer M., Schmid K., Spindler L., Ho C.H., Kreim N., Andrade-Navarro M.A., Poeck B., Helm M. (2016). m^6^A modulates neuronal functions and sex determination in *Drosophila*. Nature.

[38] Molinie B., Wang J., Lim K.S., Hillebrand R., Lu Z.X., Van Wittenberghe N., Howard B.D., Daneshvar K., Mullen A.C., Dedon P. (2016). m^6^A-LAIC-seq reveals the census and complexity of the m^6^A epitranscriptome. Nat. Methods.

[39] Xiao W., Adhikari S., Dahal U., Chen Y.S., Hao Y.J., Sun B.F., Sun H.Y., Li A., Ping X.L., Lai W.Y. (2016). Nuclear m^6^A Reader YTHDC1 Regulates mRNA Splicing. Mol. Cell.

[40] Du H., Zhao Y., He J., Zhang Y., Xi H., Liu M., Ma J., Wu L. (2016). YTHDF2 destabilizes m^6^A-containing RNA through direct recruitment of the CCR4-NOT deadenylase complex. Nat. Commun..

[41] Ivanova I., Much C., Di Giacomo M., Azzi C., Morgan M., Moreira P.N., Monahan J., Carrieri C., Enright A.J., O’Carroll D. (2017). The RNA m^6^A reader YTHDF2 is essential for the post-transcriptional regulation of the maternal transcriptome and oocyte competence. Mol. Cell.

[42] Lin S., Choe J., Du P., Triboulet R., Gregory R.I. (2016). The m^6^A Methyltransferase METTL3 promotes translation in human cancer cells. Mol. Cell.

[43] Cui Q., Shi H., Ye P., Li L., Qu Q., Sun G., Sun G., Lu Z., Huang Y., Yang C.G. (2017). m^6^A RNA methylation regulates the self-renewal and tumorigenesis of glioblastoma stem cells. Cell Rep..

[44] Zhang S., Zhao B.S., Zhou A., Lin K., Zheng S., Lu Z., Chen Y., Sulman E.P., Xie K., Bogler O. (2017). m^6^A Demethylase ALKBH5 maintains tumorigenicity of glioblastoma stem-like cells by sustaining FOXM1 expression and cell proliferation program. Cancer Cell.

[45] Xi Z., Xue Y., Zheng J., Liu X., Ma J., Liu Y. (2016). WTAP expression predicts poor prognosis in malignant glioma patients. J. Mol. Neurosci..

[46] Dominissini D., Moshitch-Moshkovitz S., Salmon-Divon M., Amariglio N., Rechavi G. (2013). Transcriptome-wide mapping of *N*^6^-methyladenosine by m^6^A-seq based on immunocapturing and massively parallel sequencing. Nat. Protocols.

[47] Bansal H., Yihua Q., Iyer S.P., Ganapathy S., Proia D.A., Penalva L.O., Uren P.J., Suresh U., Carew J.S., Karnad A.B. (2014). WTAP is a novel oncogenic protein in acute myeloid leukemia. Leukemia.

[48] Yi C., Pan T. (2011). Cellular dynamics of RNA modification. Acc. Chem. Res..

[49] Kwok C.T., Marshall A.D., Rasko J.E., Wong J.J. (2017). Genetic alterations of m^6^A regulators predict poorer survival in acute myeloid leukemia. J. Hematol. Oncol..

[50] Xiang Y., Laurent B., Hsu C.H., Nachtergaele S., Lu Z., Sheng W., Xu C., Chen H., Ouyang J., Wang S. (2017). RNA m^6^A methylation regulates the ultraviolet-induced DNA damage response. Nature.

[51] Hongay C.F., Orr-Weaver T.L. (2011). *Drosophila* Inducer of MEiosis 4 (IME4) is required for Notch signaling during oogenesis. Proc. Natl. Acad. Sci. USA.

[52] Vu L.P., Pickering B.F., Cheng Y., Zaccara S., Nguyen D., Minuesa G., Chou T., Chow A., Saletore Y., MacKay M. (2017). The *N*^6^-methyladenosine (m^6^A)-forming enzyme METTL3 controls myeloid differentiation of normal hematopoietic and leukemia cells. Nat. Med..

[53] Chen M., Wei L., Law C.T., Tsang F.H., Shen J., Cheng C.L., Tsang L.H., Ho D.W., Chiu D.K., Lee J.M. (2018). RNA *N*^6^-methyladenosine methyltransferase-like 3 promotes liver cancer progression through YTHDF2-dependent posttranscriptional silencing of SOCS2. Hepatology.

[54] Spitale R.C., Flynn R.A., Zhang Q.C., Crisalli P., Lee B., Jung J.W., Kuchelmeister H.Y., Batista P.J., Torre E.A., Kool E.T. (2015). Structural imprints in vivo decode RNA regulatory mechanisms. Nature.

[55] Liu N., Zhou K.I., Parisien M., Dai Q., Diatchenko L., Pan T. (2017). *N*^6^-methyladenosine alters RNA structure to regulate binding of a low-complexity protein. Nucleic Acids Res..

[56] Lareau L.F., Brenner S.E. (2015). Regulation of splicing factors by alternative splicing and NMD is conserved between kingdoms yet evolutionarily flexible. Mol. Biol. Evol..

[57] Bartel F., Taubert H., Harris L.C. (2002). Alternative and aberrant splicing of MDM2 mRNA in human cancer. Cancer Cell.

[58] Bohlman S., Manfredi J.J. (2014). p53-independent effects of Mdm2. Sub-Cell. Biochem..

[59] Steinman H.A., Burstein E., Lengner C., Gosselin J., Pihan G., Duckett C.S., Jones S.N. (2004). An alternative splice form of Mdm2 induces p53-independent cell growth and tumorigenesis. J. Biol. Chem..

[60] Fry N.J., Law B.A., Ilkayeva O.R., Holley C.L., Mansfield K.D. (2017). *N*^6^-methyladenosine is required for the hypoxic stabilization of specific mRNAs. RNA.

[61] Wang X., Lu Z., Gomez A., Hon G.C., Yue Y., Han D., Fu Y., Parisien M., Dai Q., Jia G. (2014). *N*^6^-methyladenosine-dependent regulation of messenger RNA stability. Nature.

[62] Shi H., Wang X., Lu Z., Zhao B.S., Ma H., Hsu P.J., Liu C., He C. (2017). YTHDF3 facilitates translation and decay of *N*^6^-methyladenosine-modified RNA. Cell Res..

[63] Fu Y., Dominissini D., Rechavi G., He C. (2014). Gene expression regulation mediated through reversible m^6^A RNA methylation. Nat. Rev. Genet..

[64] Arhin G.K., Boots M., Bagga P.S., Milcarek C., Wilusz J. (2002). Downstream sequence elements with different affinities for the hnRNP H/H’ protein influence the processing efficiency of mammalian polyadenylation signals. Nucleic Acids Res..

[65] Alarcon C.R., Lee H., Goodarzi H., Halberg N., Tavazoie S.F. (2015). *N*^6^-methyladenosine marks primary microRNAs for processing. Nature.

[66] Zheng X., Chopp M., Lu Y., Buller B., Jiang F. (2013). MiR-15b and miR-152 reduce glioma cell invasion and angiogenesis via NRP-2 and MMP-3. Cancer Lett..

[67] Chung H.J., Choi Y.E., Kim E.S., Han Y.H., Park M.J., Bae I.H. (2015). miR-29b attenuates tumorigenicity and stemness maintenance in human glioblastoma multiforme by directly targeting BCL2L2. Oncotarget.

