# Supplementary material for: N6-Methyladenosine Landscape of Glioma Stem-Like Cells: METTL3 Is Essential for the Expression of Actively Transcribed Genes and Sustenance of the Oncogenic Signaling
Source: Genes (Basel). 2019 Feb 13;10(2):141. doi: 10.3390/genes10020141 (PMC6410051; doi:10.3390/genes10020141)
Supplement: Supplementary file 1 [file genes-10-00141-s001.zip › Visvanathan et al., 2018 supplementary figures and figure legends.pdf]

Supplementary information (Visvanathan et al., 2018)

Supplementary figure legends **2**

Supplementary figure **8**

Supplementary figure legends

Supplementary Figure 1. m6A peak distribution

A. The percentage of methylated genes that carry 1, 2, 3 or 4+ peaks per gene was plotted. Most methylated genes carry only one m6A peak. **B**. Correlation analysis between m6A peak enrichment score in shNT-GSCs and number of m6A consensus sites of genes is depicted as scatter plot. P value >0.05. **C**. Percentage reduction in the m6A peaks post METTL3 silencing at different segments of transcript. **D**. Peak density distribution of m6A modification 1000 bases up- and down-stream of stop codon. **E.** Pie chart presenting the fraction for various types of RNAs differentially regulated with significance post-METTL3 silencing. The various types included are Protein-coding RNAs, lncRNA, Pseudogenes, snRNAs, snoRNAs, miRNAs and other RNAs. The lncRNA includes lincRNA, macro-lncRNA, bidirectional promoter lncRNA, sense_intronic and sense_overlapping. Pseudogenes includes pseudogene, processed pseudogenes, polymorphic pseudogenes, transcribed processed pseudogenes, transcribed unprocessed pseudogenes, transcribed unitary pseudogenes, unitary pseudogenes, unprocessed pseudogenes, TR_V pseudogene and IG_V pseudogene. Other RNAs include scaRNA, antisense, IG_V_gene, IG_C_gene, TR_V_gene, rRNA, TR_C_gene, TR_J_gene, processed transcript, misc_RNA, 3'_overlapping_ncRNA, to be experimentally confirmed and noncoding RNAs. **F**.Scatter plot for number of peak per gene showing negative correlation with differential transcript regulation post METTL3 silencing. Genes with high number of peaks show down-regulation after METTL3 inhibition.

Supplementary Figure 2. GSC specific histone activated genes relate to METTL3 direct targets

A. GSEA enrichment plots showing a negative enrichment of ACTIVE_ON_GSC_NSC gene set in METTL3 regulated direct targets (shMETTL3-shNT). This gene set consists of genes that are activated by H3K4me3 (active mark) in MGG8 GSCs but repressed by H3K27me (repression mark) or a bivalent mark (repression mark) in NSCs. **B**. The normalized read counts of transcripts (shNT-GSC and shMETTL3-GSC) that were enriched for ACTIVE_ON_GSC_NSC gene set is depicted as heat map. Red and green denotes high and low expression respectively. Note that the majority of genes of this gene set are downregulated in METTL3 silenced GSCs. **C**. GSEA enrichment plots showing a negative enrichment of ACTIVE_ON_GSC_DGC gene set in METTL3 regulated direct targets (shMETTL3-shNT). This gene set consists of genes that are activated by H3K4me3 (active mark) in MGG8 GSCs but repressed by H3K27me (repression mark) or a bivalent mark (repression mark) in MGG8 DGCs. **D**. The normalized read counts of genes (shNT-GSC and shMETTL3-GSC) that were enriched for ACTIVE_ON_GSC_DGC gene set is depicted as heat map. Red and green denotes high and low expression respectively. Note that the majority of genes of this gene set are downregulated in METTL3 silenced GSCs **E**. GSEA enrichment plots showing negative significant enrichment of ACTIVE_ON_ GSC_DGC_H3K27AC gene set in METTL3 regulated direct targets (shMETTL3-shNT). This gene set consists of genes that are activated by H3K27Ac in MGG8 GSCs and lost in MGG8 DGCs. **F**. The

normalized read counts of genes (shNT-GSC and shMETTL3-GSC) that were enriched for ACTIVE_ON_GSC_DGC_H3K27AC was depicted as heat map. Red and green denotes high and low expression respectively. Note that the majority of genes of this gene set are downregulated in METTL3 silenced GSCs

Supplementary Figure 3. GSC specific histone activated genes relate to METTL3 indirect targets

A. GSEA enrichment plots showing a negative enrichment of ACTIVE_ON_GSC_NSC gene set in METTL3 regulated indirect targets (shMETTL3-shNT). This gene set consists of genes that are activated by H3K4me3 (active mark) in MGG8 GSCs but repressed by H3K27me (repression mark) or a bivalent mark (repression mark) in NSCs. **B**. The normalized read counts of genes (shNT-GSC and shMETTL3-GSC) that were enriched for ACTIVE_ON_GSC_NSC gene set is depicted as heat map. Red and green denotes high and low expression respectively. Note that the majority of genes of this gene set are downregulated in METTL3 silenced GSCs. **C**. GSEA enrichment plots showing a negative enrichment of ACTIVE_ON_GSC_DGC gene set in METTL3 regulated indirect targets (shMETTL3-shNT). This gene set consists of genes that are activated by H3K4me3 (active mark) in MGG8 GSCs but repressed by H3K27me (repression mark) or a bivalent mark (repression mark) in MGG8 DGCs. **D**. The normalized read counts of genes (shNT-GSC and shMETTL3-GSC) that were enriched for ACTIVE_ON_GSC_DGC gene set is depicted as heat map. Red and green denotes high and low expression respectively. Note that the majority of genes of this gene set are downregulated in METTL3 silenced GSCs. **E**. GSEA enrichment plots showing negative significant enrichment of ACTIVE_ON_ GSC_DGC_H3K27AC gene set in METTL3 regulated indirect targets (shMETTL3-shNT). This gene set consists of genes that are activated by H3K27Ac in MGG8 GSCs and lost in MGG8 DGCs. **F**. The normalized read counts of genes (shNT-GSC and shMETTL3-GSC) that were enriched for ACTIVE_ON_GSC_DGC_H3K27AC was depicted as heat map. Red and green denotes high and low expression respectively. Note that the majority of genes of this gene set are downregulated in METTL3 silenced GSCs

Supplementary Figure 4. Distribution of METTL3 dependent RNA editing events

A. The event distribution of all editing events across Alu, non-Alu and non-Alu–non repeats segments for shNT and shMETTL3 conditions were depicted as pie diagram. The area of diagram represents the total number of events. **B.** The

event distribution of all editing events across different RNA segments for shNT and shMETTL3 conditions were depicted as pie diagram. The area of diagram represents the total number of events. **C**. The event distribution of all editing events across different RNA segments in shNT and shMETTL3 conditions were depicted as bar graph. The events in shNT were normalized to 100%. **D.** The amino acid alteration which leads to synonymous and non- synonymous editing events in shNT and shMETTL3 cells is plotted. **E**. Pie chart representing the rna editing events in ShNT MGG8 GSCs. The A-I event type include A-G and T-C events. C-U events include C-T and G-A events

Supplementary Figure 5. METTL3 and m6A modification in RNA processing

A. Pathway analysis of all putative RBPs identified by RBPmap and the p value of enrichment are depicted. **B**. Pathway analysis of gene groups which show aberrant alternative splicing under exon skipping (SE), retained intron (RI) and alternative splice sites (A3'SS, A5'SS) categories. **C.** Cumulative distribution of genes which show overlapping m6A peaks with skipped exon coordinates were depicted for m6A modified genes compared to non-m6A genes.

Supplementary Figure 6. METTL3 regulate the exon skipping of MDM2

The functional protein domains of MDM2 are presented. NLS, nuclear localization signal; NES, nuclear export signal; Zn-finger, zinc-finger domain; NoLS, nucleolar localization signal; RING-finger, ring-finger domain. Skipped exon events identified in shNT-GSCs that were lost in shMETTL3-GSCs were given below.

Supplementary Figure 7. Regulation of lincRNAs and miRNAs by METTL3 in GSCs

A. Volcano plot representing lincRNAs regulated after METTL3 silencing (shMETTL3-shNT) was illustrated. The significant lincRNAs regulated were marked red (upregulated) and green (downregulated) dots. METTL3 silencing resulted in an overall upregulation of lincRNAs. **B**. Heat map depicting the differential regulation of lincRNAs between shNT and shMETTL3 GSCs in duplicates was shown. Red indicates high and green for low expression in the scale of 1.5 to -1.5. **C**. Cumulative fraction of absolute abundance of transcripts in FPKM is plotted for lincRNAs and protein coding genes. Protein coding genes is higher in abundance compared to lincRNAs. **D**. Cumulative fraction of absolute abundance of transcripts in FPKM is plotted for modified and unmodified protein coding genes and lincRNAs. m6A modified protein coding genes and lincRNAs expression is higher compared to protein coding genes and lincRNAs with no m6A peak. **E**. Cumulative fraction of fold change (shMETTL3-shNT) for protein coding genes and lincRNAs that are methylated or unmethylated was shown. Both methylated lincRNAs and protein-coding genes are significantly inhibited by METTL3 silencing compared to unmethylated transcripts after METTL3 silencing. **F**. m6A peak distribution for top direct lncRNA targets XIST, MALAT1 and H19 were shown in shNT and shMETTL3 conditions. Normalized sequence coverage of RIP was indicated above the gene architecture in UCSC format. Thick black boxes represent
the coding sequences and thin lines represent introns. **G.** Heat map illustrating the differential regulated miRNAs between shNT and shMETTL3 GSCs in duplicate samples. Red indicates high and green for low expression with the scale of 2 to -2. **H**. The genes with m6A peaks at 3’UTR and downregulated post METTL3 silencing were analyzed for target miRNA binding sites within the peaks. Genes with maximum number of miRNA sites with m6A peaks were identified by target scan and the network was visualized by Cytoscape. miRNAs that are upregulated in GBM compared to control brain were marked red.

Supplementary Figure 8. Pathways regulated by direct and indirect METTL3 targets

A and B. PANTHER pathways enriched in direct and indirect targets were plotted respectively. The top 10 pathway terms, which were enriched with significant p value of less than 0.05, were depicted.

Supplementary Figure 9. Regulation of NOTCH pathway by METTL3 mediated m^{6}a modification

A. Gene Set Enrichment Analysis (GSEA) was performed for genes which are regulated by METTL3 at transcript level with 2-fold regulation ($p<0.05$). The gene set - NGUYEN_NOTCH1_TARGETS_UP is negatively enriched in METTL3 silenced GSCs. **B**. Table representing the regulation of NOTCH pathway genes upon METTL3 silencing and the m^{6}a modification .

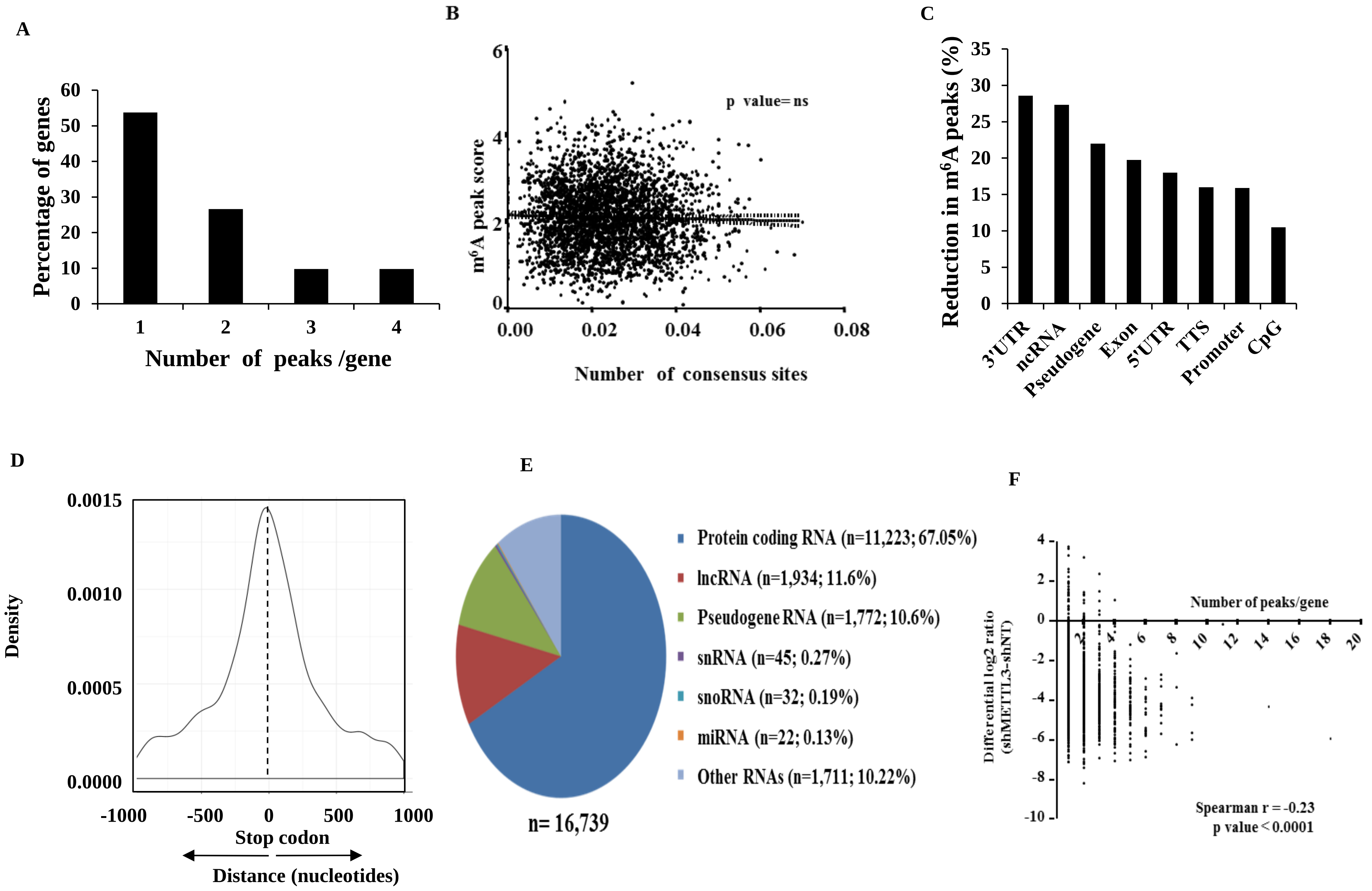
A
Percentage of genes
60
50
40
30
20
10
0
1
2
3
4
Number of peaks /gene
B
m6A peak score
6
4
2
0
0.00
0.02
0.04
0.06
0.08
Number of consensus sites
p value= ns
C
Reduction in m6A peaks (%)
35
30
25
20
15
10
5
0
3'UTR
ncRNA
Pseudogene
Exon
5'UTR
TTS
Promoter
CpG
D
Density
0.0015
0.0010
0.0005
0.0000
-1000
-500
0
500
1000
Stop codon
Distance (nucleotides)
E
Protein coding RNA (n=11,223; 67.05%)
lncRNA (n=1,934; 11.6%)
Pseudogene RNA (n=1,772; 10.6%)
snRNA (n=45; 0.27%)
snoRNA (n=32; 0.19%)
miRNA (n=22; 0.13%)
Other RNAs (n=1,711; 10.22%)
n= 16,739
F
Differential log2 ratio (shMETTL3-shNT)
Number of peaks/gene
Spearman r = -0.23
p value < 0.0001

Supplementary figure 2; Visvanathan et al., 2018

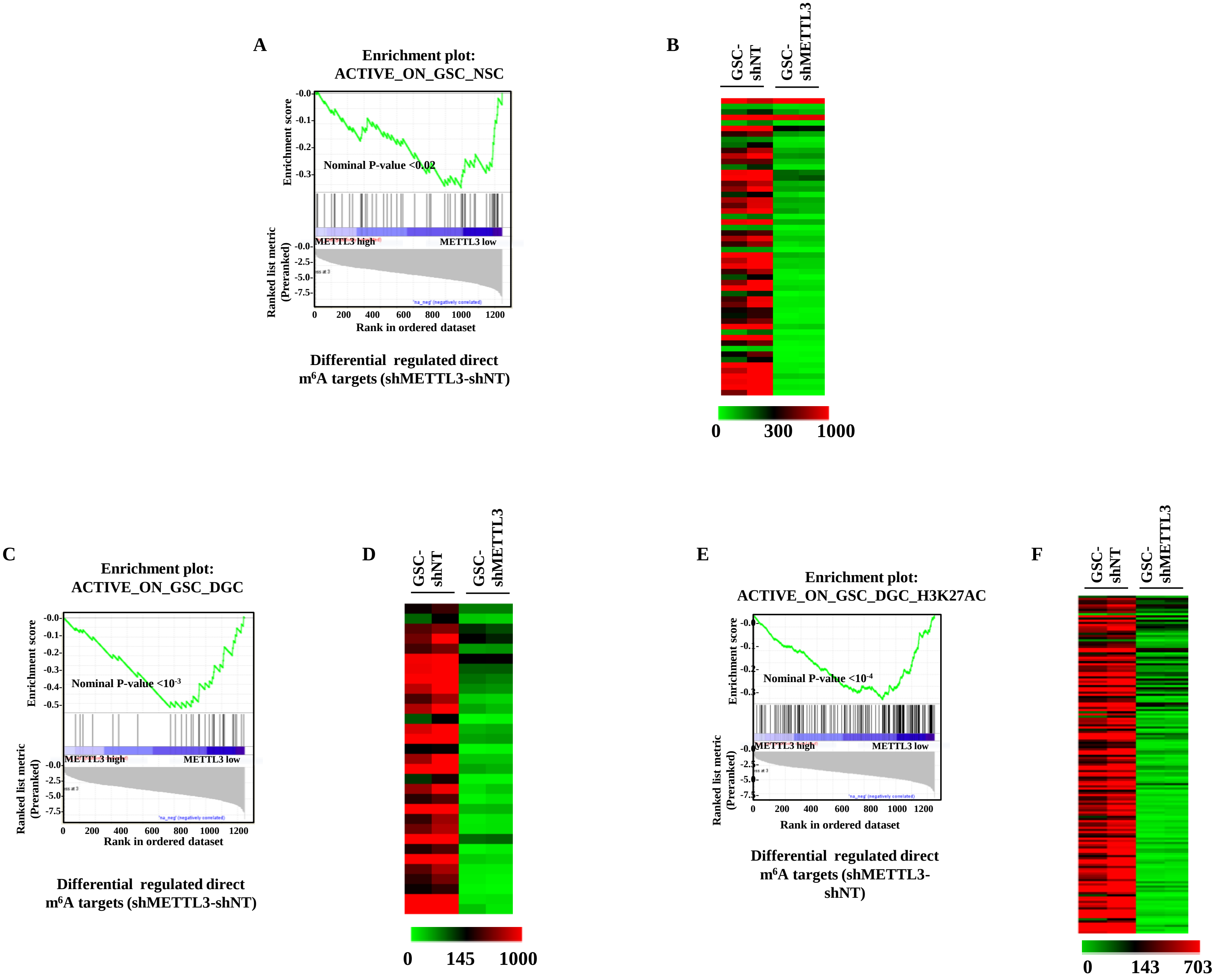

Supplementary figure 3; Visvanathan et al., 2018

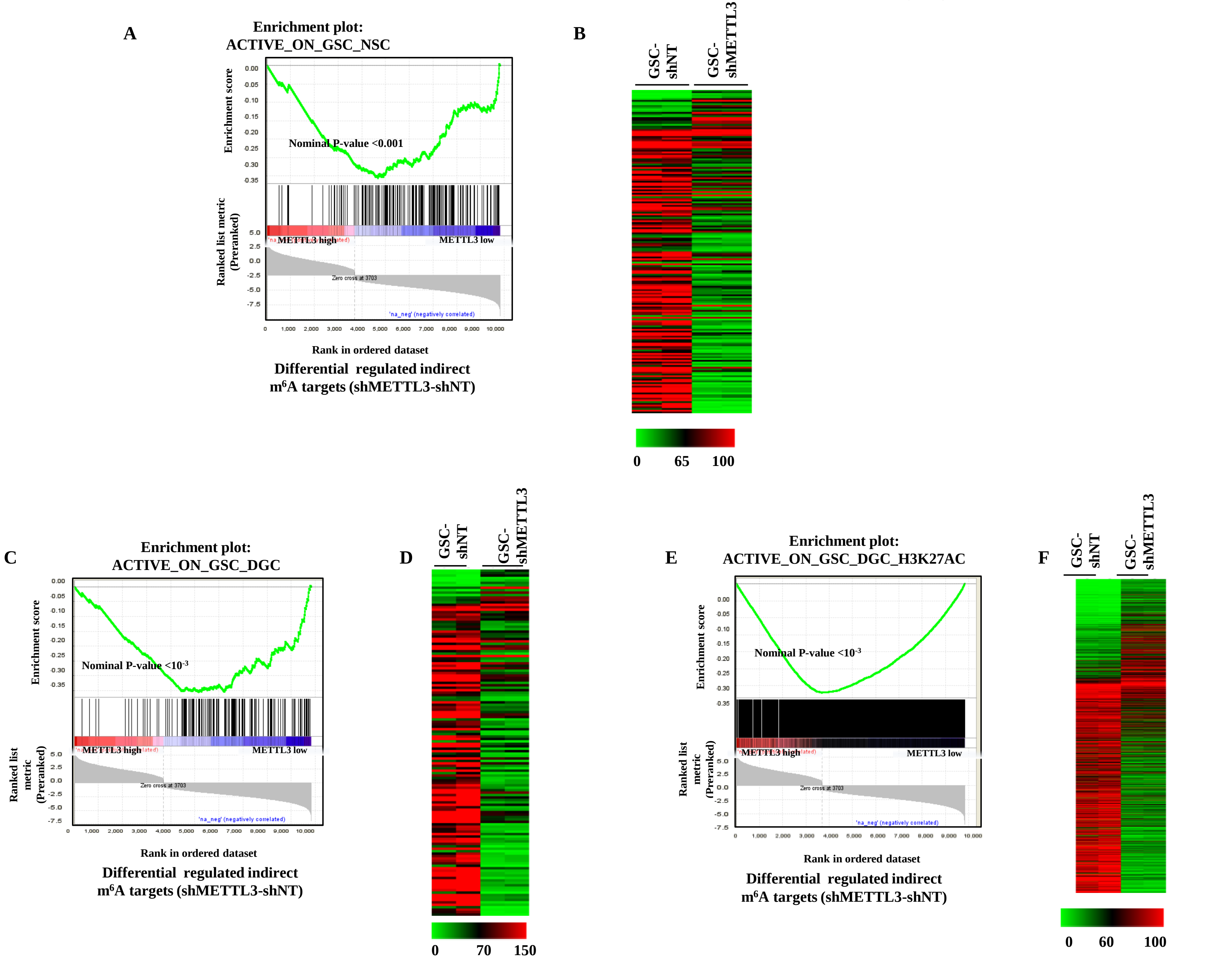

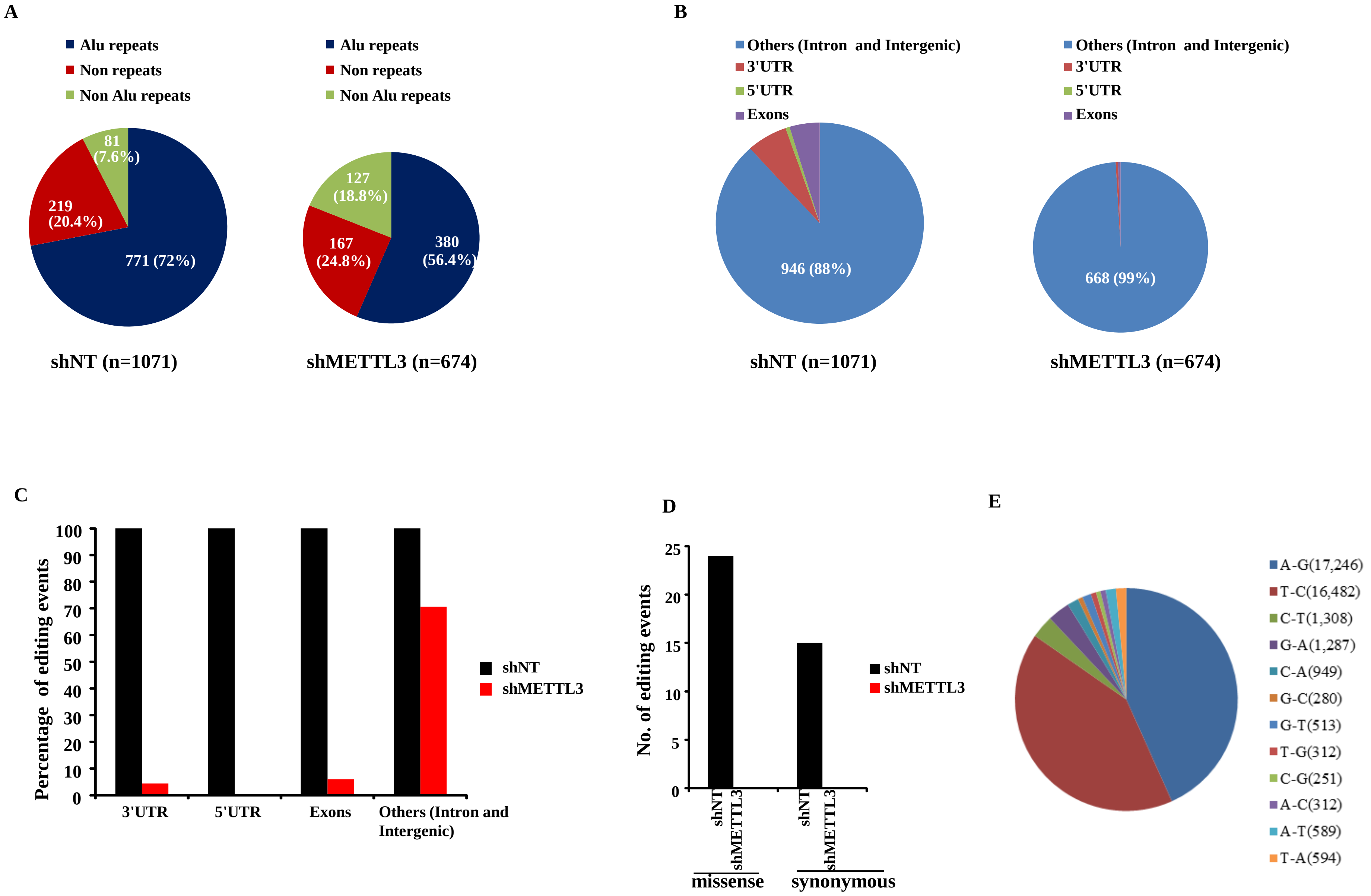
A
Alu repeats
Non repeats
Non Alu repeats
81 (7.6%)
219 (20.4%)
771 (72%)
127 (18.8%)
167 (24.8%)
380 (56.4%)
shNT (n=1071)
shMETTL3 (n=674)
B
Others (Intron and Intergenic)
3'UTR
5'UTR
Exons
946 (88%)
668 (99%)
shNT (n=1071)
shMETTL3 (n=674)
C
Percentage of editing events
100
90
80
70
60
50
40
30
20
10
0
3'UTR
5'UTR
Exons
Others (Intron and Intergenic)
shNT
shMETTL3
D
No. of editing events
25
20
15
10
5
0
shNT
shMETTL3
missense
synonymous
E
A-G(17,246)
T-C(16,482)
C-T(1,308)
G-A(1,287)
C-A(949)
G-C(280)
G-T(513)
T-G(312)
C-G(251)
A-C(312)
A-T(589)
T-A(594)

Supplementary figure 5; Visvanathan et al., 2018

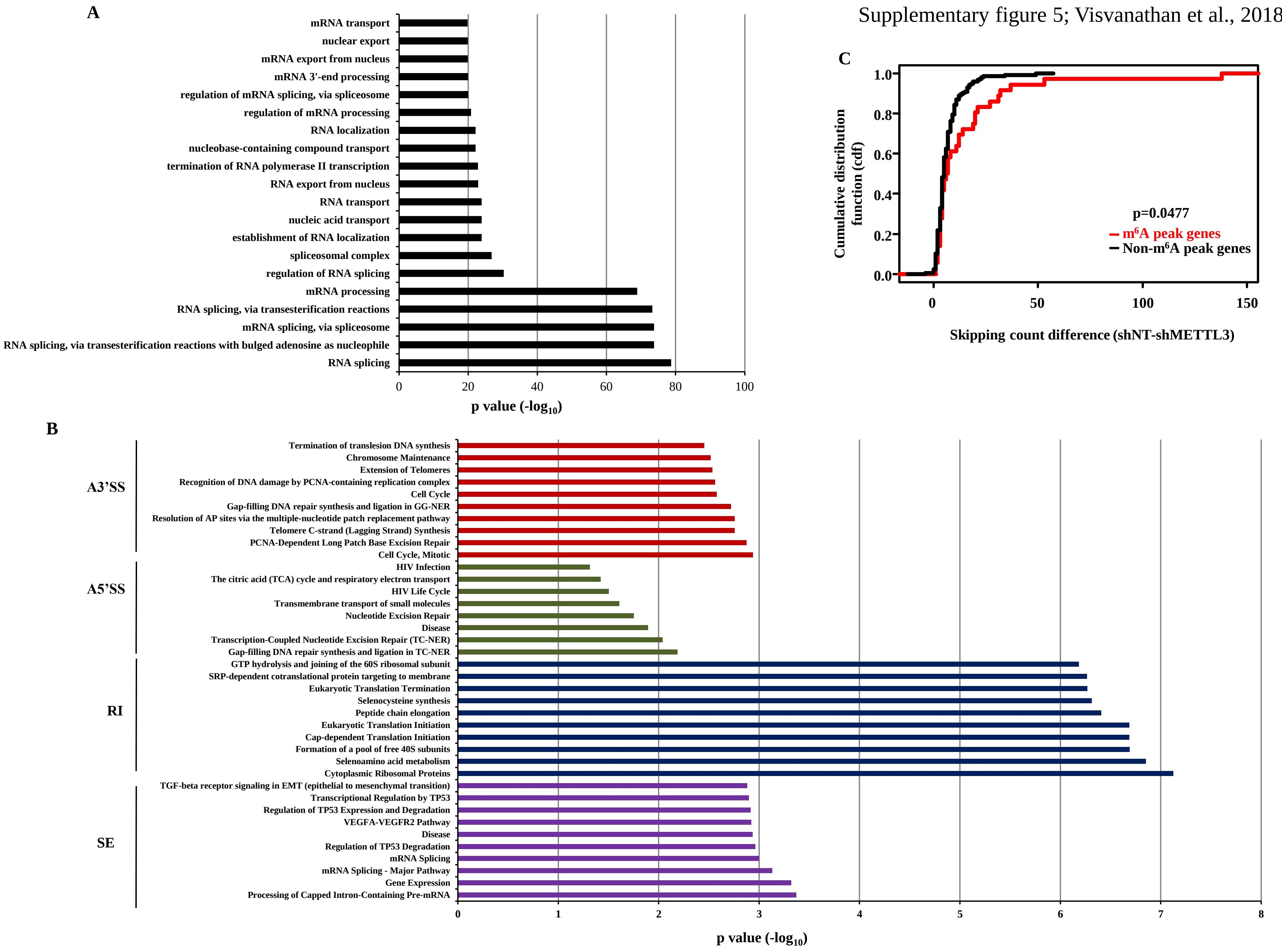

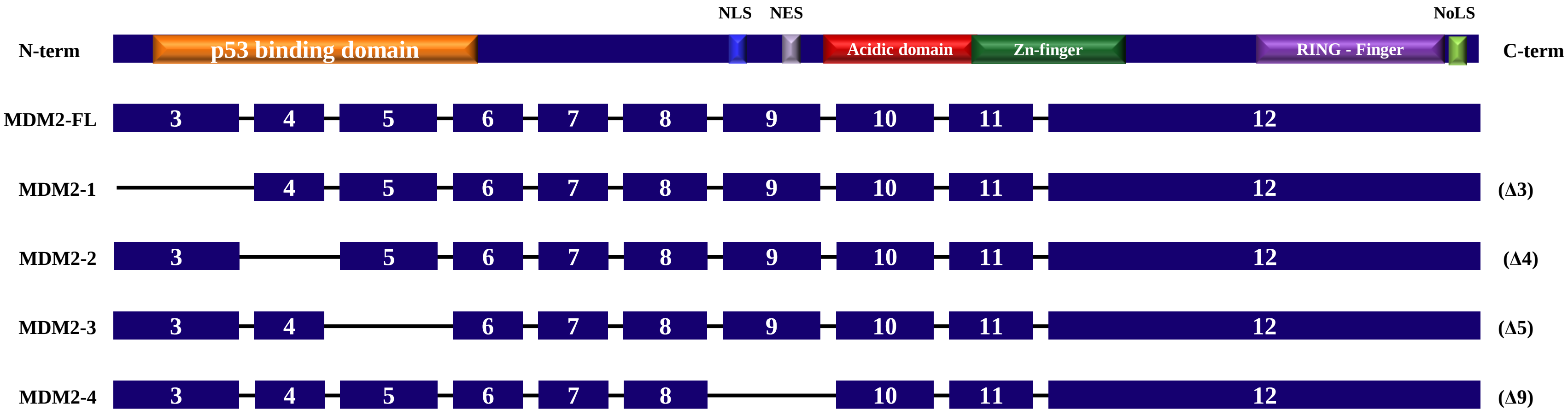
NLS
NES
NoLS
N-term
p53 binding domain
Acidic domain
Zn-finger
RING - Finger
C-term
MDM2-FL
3 4 5 6 7 8 9 10 11 12
MDM2-1
4 5 6 7 8 9 10 11 12
(Δ3)
MDM2-2
3 5 6 7 8 9 10 11 12
(Δ4)
MDM2-3
3 4 6 7 8 9 10 11 12
(Δ5)
MDM2-4
3 4 5 6 7 8 10 11 12
(Δ9)

MDM2

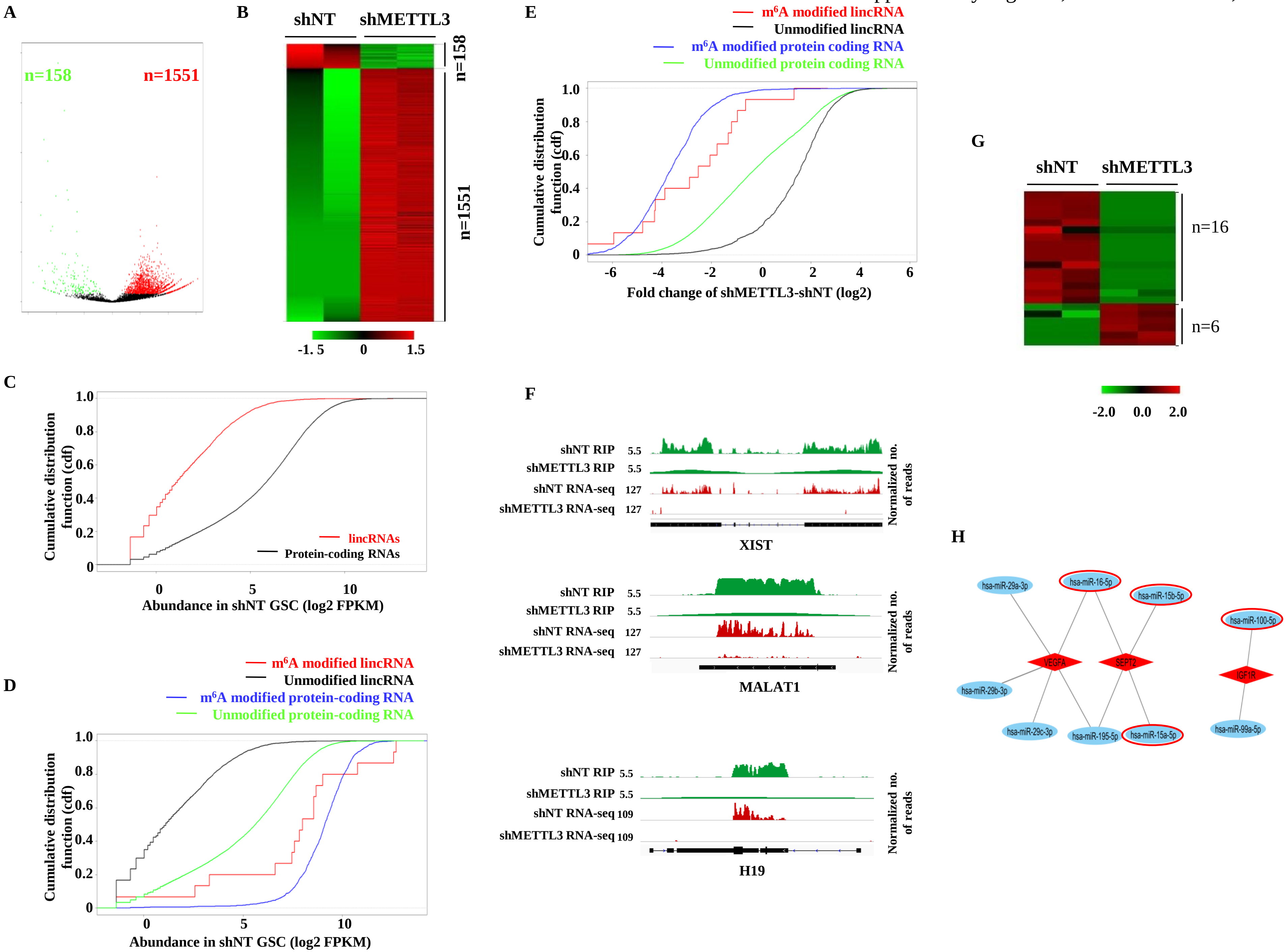
A
n=158
n=1551
B
shNT
shMETTL3
n=158
n=1551
-1. 5
0
1.5
C
Cumulative distribution function (cdf)
lincRNAs
Protein-coding RNAs
Abundance in shNT GSC (log2 FPKM)
D
m6A modified lincRNA
Unmodified lincRNA
m6A modified protein-coding RNA
Unmodified protein-coding RNA
Cumulative distribution function (cdf)
Abundance in shNT GSC (log2 FPKM)
E
m6A modified lincRNA
Unmodified lincRNA
m6A modified protein coding RNA
Unmodified protein coding RNA
Cumulative distribution function (cdf)
Fold change of shMETTL3-shNT (log2)
F
shNT RIP 5.5
shMETTL3 RIP 5.5
shNT RNA-seq 127
shMETTL3 RNA-seq 127
Normalized no. of reads
XIST
MALAT1
shNT RIP 5.5
shMETTL3 RIP 5.5
shNT RNA-seq 109
shMETTL3 RNA-seq 109
H19
G
shNT
shMETTL3
n=16
n=6
-2.0
0.0
2.0
H
hsa-miR-29a-3p
hsa-miR-16-5p
hsa-miR-15b-5p
hsa-miR-100-5p
VEGFA
SEPT2
IGF1R
hsa-miR-29b-3p
hsa-miR-29c-3p
hsa-miR-195-5p
hsa-miR-15a-5p
hsa-miR-99a-5p

A

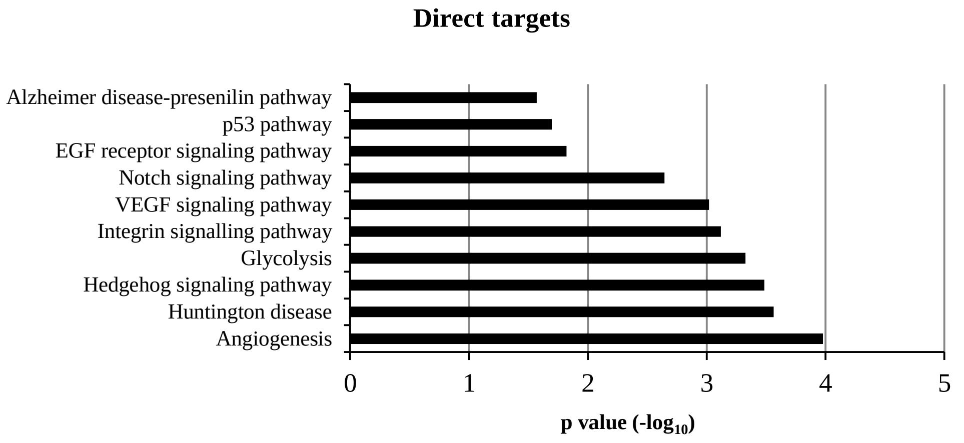
Direct targets
Alzheimer disease-presenilin pathway
p53 pathway
EGF receptor signaling pathway
Notch signaling pathway
VEGF signaling pathway
Integrin signalling pathway
Glycolysis
Hedgehog signaling pathway
Huntington disease
Angiogenesis
0
1
2
3
4
5
p value (-log10)

B

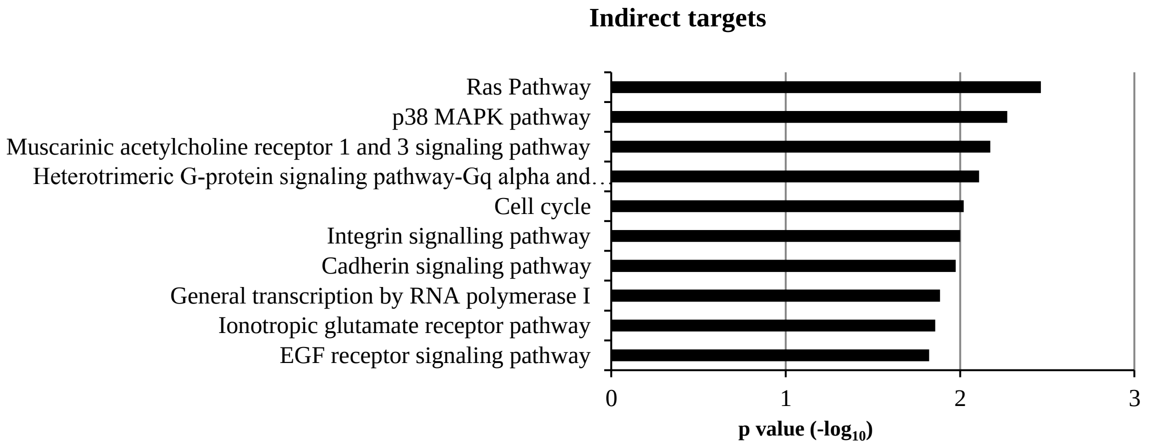
Indirect targets
Ras Pathway
p38 MAPK pathway
Muscarinic acetylcholine receptor 1 and 3 signaling pathway
Heterotrimeric G-protein signaling pathway-Gq alpha and…
Cell cycle
Integrin signalling pathway
Cadherin signaling pathway
General transcription by RNA polymerase I
Ionotropic glutamate receptor pathway
EGF receptor signaling pathway
0
1
2
3
p value (-log10)

A

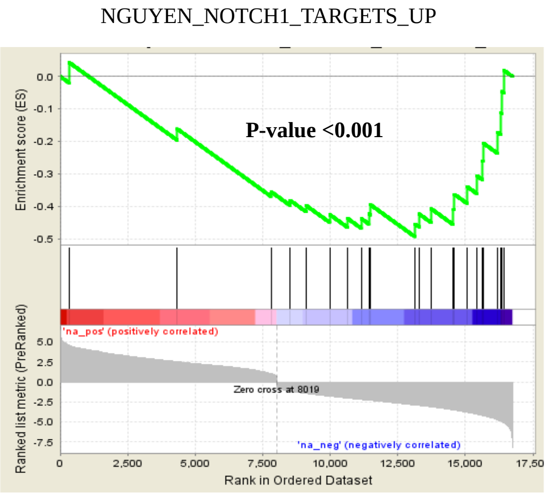

B

Regulation of Notch signaling by METTL3 dependent m6a modification					
**Gene symbol**	**Differential expression log2 (shMETTL3-shNT)**	**p value**	**p adj**	**Number of m6a peaks in shNT GSC**	**Number of m6a peaks in shMETTL3 GSC**
DLL1	-4.87718	1.34E-16	2.53E-15	1	0
DLL3	-4.98771	2.36E-27	1.04E-25	2	0
DLL4	1.181379	0.119828	0.187499	0	0
JAG1	-2.66791	4.21E-15	7.05E-14	0	0
JAG2	-4.00343	3.75E-25	1.4E-23	1	0
Notch1	-5.20051	6.78E-50	1.91E-47	2	0
Notch2	-4.25615	5.38E-45	1.05E-42	1	0
Notch3	-3.91067	2.95E-34	2.35E-32	2	0
Notch4	1.374536	0.00036	0.001511	0	0
HES1	-3.5254	1.88E-12	2.54E-11	1	0
